# Functional analysis of *SlNCED1* in pistil development and fruit set in tomato (*Solanum lycopersicum* L.)

**DOI:** 10.1038/s41598-019-52948-2

**Published:** 2019-11-15

**Authors:** Wenbin Kai, Ying Fu, Juan Wang, Bin Liang, Qian Li, Ping Leng

**Affiliations:** College of Horticulture, China Agricultural University, Beijing, 100193 China

**Keywords:** RNAi, Plant physiology

## Abstract

Abscisic acid (ABA) is an important regulator of many plant developmental processes, although its regulation in the pistil during anthesis is unclear. We investigated the role of 9-*cis*-epoxycarotenoid dioxygenase (*SlNCED1*), a key ABA biosynthesis enzyme, through overexpression and transcriptome analysis in the tomato pistil. During pistil development, ABA accumulates and *SlNCED1* expression increases continually, peaking one day before full bloom, when the maximum amount of ethylene is released in the pistil. ABA accumulation and *SlNCED1* expression in the ovary remained high for three days before and after full bloom, but then both declined rapidly four days after full bloom following senescence and petal abscission and expansion of the young fruits. Overexpression of *SlNCED1* significantly increased ABA levels and also up-regulated *SlPP2C5* expression, which reduced ABA signaling activity. Overexpression of *SlNCED1* caused up-regulation of pistil-specific Zinc finger transcription factor genes SlC3H29, SlC3H66, and SlC3HC4, which may have affected the expression of *SlNCED1*-mediated pistil development-related genes, causing major changes in ovary development. Increased ABA levels are due to *SlNCED1* overexpresson which caused a hormonal imbalance resulting in the growth of parthenocarpic fruit. Our results indicate that *SlNCED1* plays a crucial role in the regulation of ovary/pistil development and fruit set.

## Introduction

Flowering and fruiting are the most important agricultural characters in a horticultural crop. The increasing numbers of reports describing the involvement of hormones in the development of flowers and fruits indicate that abscisic acid (ABA) regulates the differentiation of floral organs and fruit ripening^1–3^. ABA functions by its concentration and through signal transduction in individual plant cells or tissues. ABA levels are determined by the processes of biosynthesis and degradation which are adjusted by 9-cis epoxycarotenoid dioxygenase (NCED) genes or *CYP707A* genes, respectively. In addition, release of ABA via a reversible reaction of Glc-conjugated ABA (ABA-GE) contributes to ABA homeostasis as well^4–6^. The ABA-mediated signaling cascade is initiated by the perception of ABA by ABA receptors, a large family of soluble PYR/PYL/RCAR (PYL) proteins^7^. Also, an ABA-PYL-PP2C (protein phosphatase-type 2 C)-SnRK2 (SNF1-related kinase 2) route is regarded as the core pathway of ABA signaling^8,9^.

ABA does not only regulate the ripening of non-climacteric fruits such as grapes and strawberries^10,11^, but it also plays a crucial role in the regulation of ripening in the climacteric tomato fruit^12–14^. ABA is also involved in early gametogenesis during reproductive development. For example, ABA affects the induction of embryogenesis and microspore development^15–18^. The overexpression of *SlNCED1*, a key ABA biosynthesis gene, affects the development and germination of pollen in tomato^3^. The pistil is a structure that is composed of the stigma, the style, and the ovary that forms heterogeneous tissues, and the development of each tissue may directly influence fruit set and subsequent fruit growth. In recent years, a large number of pistil-specific genes have been identified, and these genes are involved in pollen tube extension, pollen-pistil interactions, and ovule development. Thus, this group of genes plays an important role in the regulation of the development of specific pistil tissues^19–21^. Zinc finger proteins (ZFPs), a family of transcription factors (TFs), are known to affect plant growth and development in many ways, such as the transcriptional regulation, RNA binding, and protein-protein interactions^22^. Recently, C2H2-ZFPs were found to be indispensable regulators of floral organ morphogenesis and also pollen and pistil maturation^23^. However, it is presently unknown whether ABA is involved in the regulation of pistil development in tomato.

Fruit set is a crucial stage of development in which the ovary is transformed into a fruit^24^. During anthesis, the ovary barely expands until growth signals from the newly fertilized ovules are received. Molecular, genetic, and biochemical analyses have shown that plant hormones such as auxin and gibberellins (Gas) play important roles in ovary development during fruit set^25,26^. Pollination and fertilization can generate an auxin signal in plants to promote GA synthesis in the ovule, which is then subsequently transported to the pericarp to promote fruit set^27^. Therefore, the synergistic combination of the effects of auxin and GA may be part of a signaling process that promotes cell division and leads to fruit set after fertilization^28^. ABA is involved in fruit set as an antagonist of IAA and GAs to maintain the pre-anthesis unpollinated ovary in a temporally protected and dormant state^29^. However, the role of ABA in the regulation of fruit set still lacks molecular evidence.

In this study, the role of *SlNCED1* in the pistil was investigated using a *SlNCED1* overexpression strategy and transcriptome analysis. Our results demonstrate that *SlNCED1* plays an important role in the regulation of pistil/ovary development and fruit set in tomato.

## Materials and Methods

### Plant material and treatments

The tomato (*Solanum lycopersicum*) cultivar ‘JiaBao’, a fresh market variety, was used as the experiment material. Fruits of cv. ‘JiaBao’ are large and oblate in shape, and the plants grow strongly to a height of one meter (Fig. S1). Seeds of the wild type (WT) inbred line ‘JiaBao’ and the transgenic lines were germinated on plates at 25 °C and then grown in a climate-controlled greenhouse at 24 °C/18 °C (day/night) under natural light. For morphological observation and making tissue sections, 10 flowers in each line were labeled, and the size of the floral buds was measured and sampled at different lengths: 2 mm, 4 mm, 6 mm, 7–9 mm, and 10–12 mm (stages 6 to 13); 13–14 mm, when the buds reach their full length (stage 15); buds with separated sepals (stage 16); flowers one day before opening (stage 18); open flowers (stage 20); and 0–7 days after full bloom during fruit set. In addition, 30 floral buds were harvested at each sampling from the transgenic line or the WT plants and were divided into three groups for three biological replicates (each biological replicate has 10 flower buds). Each flower group was weighed, the ethylene level was measured, and the pistils were then separated from the flowers and sampled. All samples were immediately frozen in liquid nitrogen, powdered, mixed, and stored at −80 ^°^C until they were used for determining physiological parameters and expression of genes related to pistil development and fruit set. At growth stages 13–14, WT and overexpression (OE) line 2 pistils were sampled for RNA-seq analysis.

### Generation of *SlNCED1*-OE transgenic tomato lines

The *35S* promoter::*SlNCED1* full length::*Nos*-terminator fusion gene cassette was cloned into pCAMBIA1305.1 (Invitrogen, Carlsbad, CA, USA), and the construct was introduced into tomato plants via Agrobacterium *tumefaciens* LBA4404-mediated transformation (Specific primers are shown in Table S1). Eight independent *SlNCED1-*overexpressing (*SlNCED1-*OE) transgenic lines were obtained. Of these, *SlNCED1* OE-1 and OE-2 were selected for use in the experiments because the expression of *SlNCED1* was significantly increased in these two lines compared to the other six transgenic OE lines.

### qRT-PCR analysis

Extraction of total RNA was performed using the SV Total RNA Isolation System (Promega). Purified RNA was used as a template for first-strand cDNA synthesis with the Takara RNA PCR Kit. qRT-PCR assays were performed on the Rotor-Gene 3000 system (Corbett Research) using SYBR Premix Ex Taq (Takara Bio). Three independent samples were analyzed for every transgenic line or WT for each sampling, and the expression was normalized using the *SAND* gene (SGN-U316474, Solyc03g115810.2.) as an internal control. The primer pairs were test by PCR and the PCR product of each gene was confirmed by the agarose gel electrophoresis and sequencing. qRT-PCR analysis was performed by the “two standard curves method”: specific primers were used to clone the fragments of each target gene and reference gene, and then the PCR fragments were ligated into the pMD18-T vector for amplification, while the two standard curves were prepared by the concentration gradient dilution of plasmids. The relative expression level of each gene was calculated using the “two standard curves method” with Rotor-Gene 6.1.81 software. The value of the lower sample was set at 1. The sequences of the oligonucleotide primers used for qRT-PCR are given in Supplemental Table 2.

### Determination of IAA, ABA, and GA contents

All tissue samples were ground to a powder in liquid N_2_ using a mortar and pestle. For each sample, 3 g of powder was extracted with 40 ml 80% methanol (v/v) containing 4 mg 2,6-di-tert-butyl-4-methylphenol. The extracts were then centrifuged at 10,000 × *g* for 20 min at 4 °C, and the supernatants were evaporated at 40 °C in a rotary evaporator. The residues were solubilized by adding 10 ml petroleum ether and then 10 ml 0.02 M phosphate buffer solution (pH 8.0) to each. After the solutions were decolorised, 0.2 g insoluble PVPP (crosslinked polyvinylpyrrolidone) was added and mixed at 0 °C for 15 min. The PVPP was then removed by vacuum filtration. Ethyl acetate (pH 3.0) was added to the solutions and the upper layers were removed and evaporated to dryness at 40 °C. Each residue was dissolved in 1 ml 50% methanol (v/v) for HPLC analysis. Aliquots of each sample (20 µl) were separated by HPLC (1200 Series; Agilent Technologies, USA) on a 4.8 × 150 mm C18 column (Agilent Technologies) with a flow rate of 0.8 ml min^–1^. The solvents were 0.8% (v/v) glacial acetic acid (solvent A) and 100% methanol (solvent B). The hormones were eluted from the column using a changeable gradient of solvent B. The retention times of the hormones were determined with three commercial standards: (±)-abscisic acid (A1049, Sigma, St Louis, MO, USA), IAA (Indole-3-Acetic acid, Sigma, I2886), and GA_3_ (gibberellin A_3_, Sigma) at a wavelength of 260 nm.

### Determination of ethylene production

Ten fresh pistils were enclosed in a 50 ml airtight container for 2 h at 20 °C. Then 1 ml of the headspace gas was taken out and injected into a gas chromatograph (Agilent model 6890 N) fitted with a flame ionization detector and an activated alumina column.

### Light microscopy assays

All flower bud samples were fixed in FAA solution (70% ethanol:acetic acid:formaldehyde; 18:1:1 by volume). The fixed samples were dehydrated in a graded ethanol series, embedded in paraffin, and then sectioned at 8 µm. Serial longitudinal and transverse sections were stained with hematoxylin-fast green^30^ and were observed under a light microscope (OLYMPUS BX41, Olympus Corporation).

### ***In situ*** hybridization

Based on previous reports^31,32^, we used non-radioactively-labeled RNA for *in-situ* hybridization. Floral bud samples were fixed in FAA, dehydrated in an ethanol series, embedded in paraffin, and then cut into with 8 μm-thick sections^3^. All slides were dewaxed with Histo-Clear and then dehydrated and baked. To synthesize the probe, sense and antisense RNAs of *SlNCED1* were labeled with digoxigenin by *in vitro* transcription of linearized pSPT18-SlNCED1, a recombinant plasmid carrying the *SlNCED1* cDNA that was amplified with gene-specific forward (5′-GAATTCAGGCAACAGTGAAACTTCCATCAAG-3′) and reverse (5′-AAGCTTTCCATTAAAGAGGATATTACCGGGGAC-3′) primers. For hybridization, all slides were successively re-hydrated, treated with Proteinase K, and dehydrated. For pre-hybridization, each slide was incubated with 100 µL hybridization solution with 77.2 µL buffer A and 22.8 µL buffer B at 42 °C for 1 hour. Buffer A (77.2 µL) was made by 50 µL formamide, 10 µL 50% dextran sulfate, 10 µL 10 X block reagent, 6 µL 5 M NaCl, 1 µL 1 M Tris-HCl (pH7.5), and 0.2 µL 500 mM EDTA (pH7.5). Buffer B was made by 2.5 µL 20 µg/µl poly A, 1.5 µL 10 mg/ml tRNA. The slides were then incubated in 100 µL hybridization solution containing the digoxygenin probe (more than 500 ng/ µL) at 42 °C overnight. The next morning, the slides were washed in 4X SSC solution for 5–10 min and then in 2X SSC for 30 min. Finally, the slides were incubated with anti-digoxigenin antibodies (diluted by 10,000 times) coupled with alkaline phosphatase and then with nitro blue tetrazolium to detect the hybridization signals.

### RNA-sequencing

Total RNA was extracted from WT and *SlNCED1*-OE-2 ovaries at floral development stage 13. We used 3 μg RNA per sample for mRNA purification and library construction with the Truseq™ RNA Sample Prep Kit (Illumina, CA, USA). The samples were sequenced on an Illumina HiSeq™ 2000 instrument. Each sample yielded more than 6 Gb of data (the complete protocol is provided in^3^).

### Statistical treatment of the data

All samples included three biological replicates; the data were statistically analyzed by SPSS software using one-way analysis of variance (ANOVA) and Duncan’s test of significance. *P value t-test < 0.05; **P value t-test 0.01.

## Results

### ABA accumulation, ethylene release, and gene expression during pistil development

**Figure 1:**
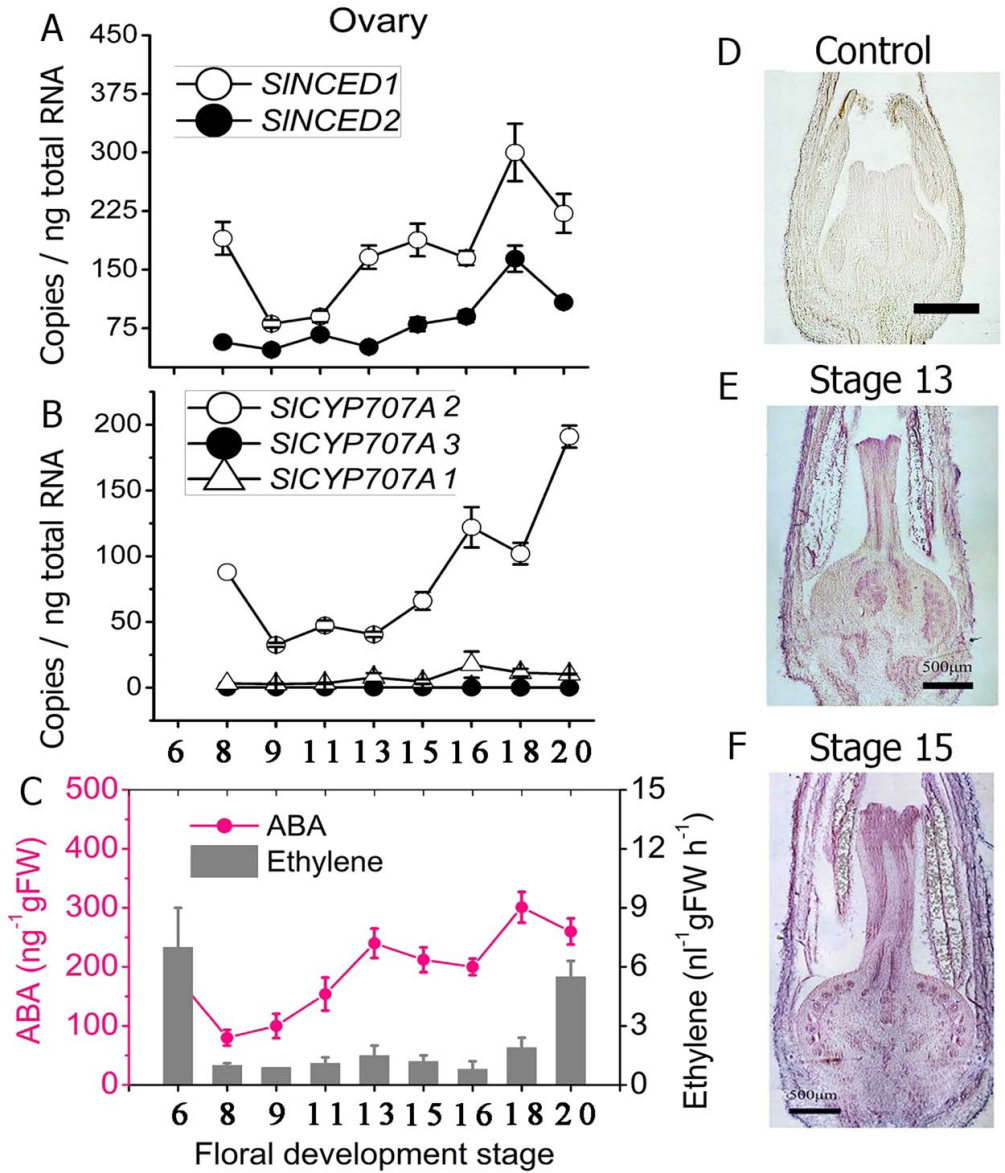
Changes in the ABA and ethylene contents and the transcriptional levels of ABA metabolism genes in the tomato (*S*. *lycopersicum* cv. ‘JiaBao’) pistil during development. **(A**,**B)** Expression of the *SlNCED* and *SlCYP707* genes. *SAND* was used as the internal control for normalization of gene expression. **(C)** ABA accumulation and ethylene release. Three biological replicates (n = 3) were used for data analysis. Error bars indicate the SE. **(D–F**) Spatio-temporal expression of *SlNCED1*. Using situ hybridization, the expression of *SlNCED1* was detected in flower buds at developmental stages 13 and 15. **(D)** Control. Scale bar = 200μm.

As previously reported^3^, we divided the flower development process into 20 stages based on Brukhin’s classification scheme^33^. As shown in Fig. 1A, we examined the expression levels of genes involved in ABA biosynthesis (*SlNCED1/2*) and catabolism (*SlCYP7071/2/3*) (Fig. 1B). The expression of *SlNCED1* was high during the early stages and then declined rapidly until stage 9, after which it increased rapidly from stage 11 and peaked one day before full bloom (DBFB) at stage 18 (Fig. 1A). The expression pattern of *SlNCED2* was similar to that of *SlNCED1* but lower during floral development. Of the *SlCYP707* genes, *SlCYP7072* was found to be highly expressed in the ovary (Fig. 1B). The expression of *SlCYP7072* was high at stage 6 and then declined, but it increased again at stage 15 and peaked at the full bloom stage. Expression levels of *SlCYP7071/3* were very low throughout flower development. In the ovary, starting from stage 8, the ABA content increased gradually, and reached its maximum value at 1 DBFB (Fig. 1C). In addition, high ethylene release was detected in the pistil at stage 6, after which it rapidly declined, until increasing again during full bloom at stage 20 (Fig. 1C). These results show that ABA levels and ethylene release are relatively high in the ovary at full bloom. We next chose two typical stages of flower development for *in situ* hybridization analysis to further examine the temporospatial expression patterns of *SlNCED1*. Figure 1D shows the control. As shown by the red stain color in Fig. 1E, *SlNCED1* showed strong expression in pollen grains and the pistil, stigma, ovules, and style at stage 13. At stage 15, there was no *SlNCED1* expression detected in the pollen grains, which appeared yellow and transparent. However, *SlNCED1* was highly expressed largely in the pistil, stigma, ovules and style (Fig. 1F). In addition, we examined the expression levels of genes involved in the ABA signaling, ethylene synthesis and the signaling in WT ovary during development (Figs S2, S3).

**Figure 2:**
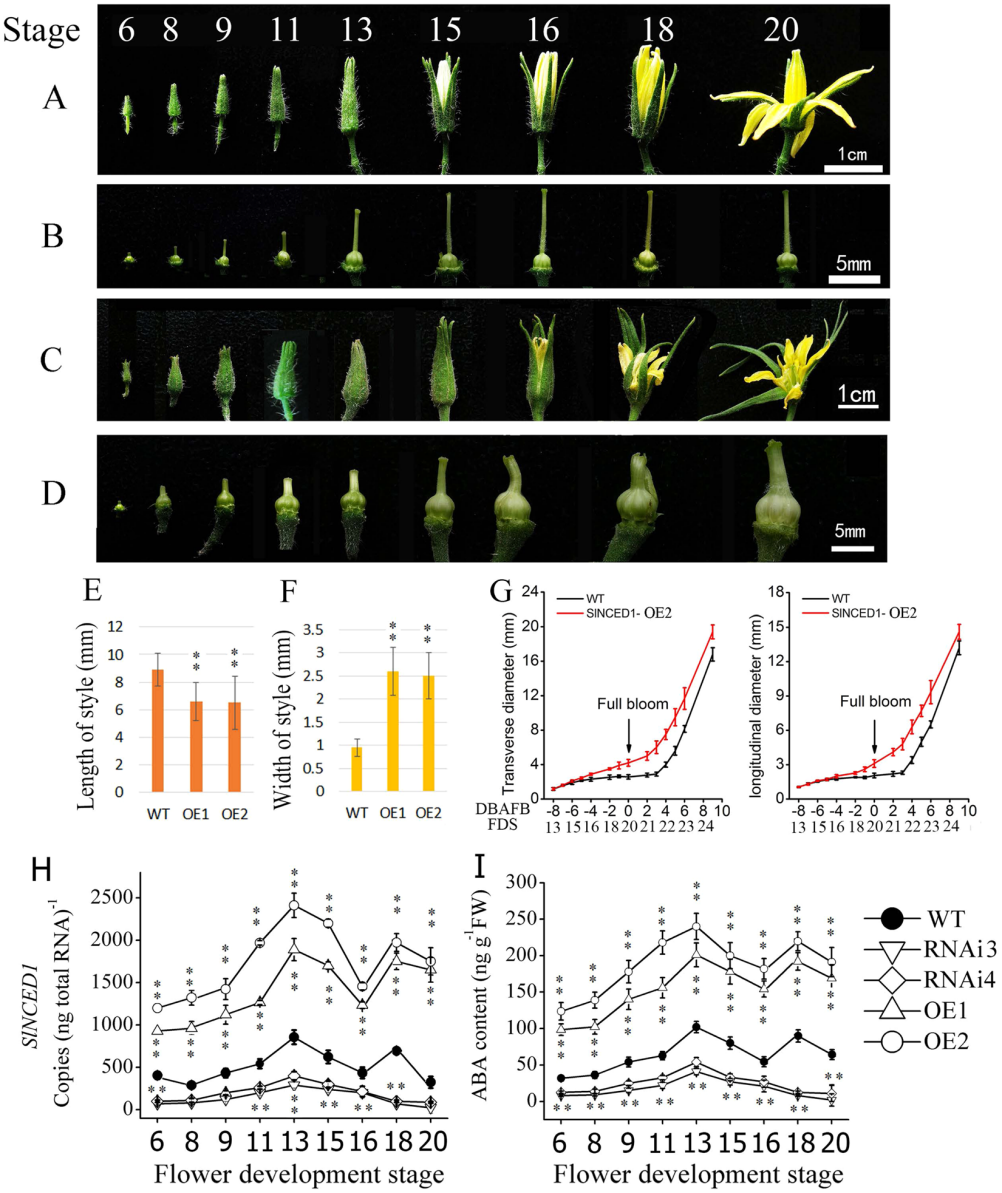
Changes in flower morphology in the WT and the *SlNCED1-OE*-2 transgenic line during floral development. **(A)** Changes in WT (cv. ‘JiaBao’) flower morphology from developmental stages 6 to 20. **(B)** The flowers shown in (**A**) with the sepals and petals removed to show the pistils. **(C)** Changes in flower morphology in the *SlNCED1-OE*-2 transgenic tomato line. **(D)** Flowers shown in (**C**) with the sepals and petals removed to show the pistils. **(E)** Style length and **(F)** Style width in the WT and two *SlNCED1*-OE lines during full bloom. **(G)** Comparisons of the transverse and longitudinal diameters of the ovary before and after full bloom. Three biological replicates (15 flowers) were used for each analysis. Error bars indicate the SE. **(H)** *SlNCED1* gene expression and **(I)** ABA accumulation in pistils of *SlNCED1-OE*-1 and -2 and *SlNCED1-*RNAi-3 and -4 lines in floral development stages 6 to 20. *SAND* (SGN-U316474) was used as the internal control for gene expression normalization. Three biological replicates (n = 3) were used for each analysis. Error bars indicate the SE. *P-value t-test < 0.05; **P-value t-test < 0.01.

### Effect of *SlNCED1* overexpression during ovary development

According to Brukhin *et al*.^33^, after the emergence of the sepal, petal, and stamen primordia, the pistil meristem appears at stages 4–5. The central column and style, and subsequently the ovule primordia develop at stages 7–8. Megasporocyte meiosis occurs at stage 11–12. The embryo sac develops as the functional megaspore undergoes mitosis at stage 14, and its formation is an asynchronous process that can last from stage 14 until stage 18. At stage 16, the sepals are better separated, and the faint yellow petals are more clearly visible. The style reaches its maximum size and ceases to grow at stage 18. Stage 18 is 1 DBFB, and the petals begin to unroll. Stage 20 is defined as a fully open flower (full bloom). Following, we defined the young fruit development stages (FDS) as 21 (1–3 days after full bloom, DAFB), 22 (4 DAFB), 23 (6–7 DAFB), and 24 (8 DAFB).

Because *SlNCED1* is the key biosynthetic gene which determines ABA levels in the ovary during flower development, we overexpressed *SlNCED1* in an effort to increase the endogenous ABA levels. A *SlNCED1*-OE vector was constructed in which the gene is under the transcriptional control of the *35S* promoter, and this overexpression construct was transferred into tomato via agrobacterium-mediated transformation. Eight independent *SlNCED1*-OE lines were obtained in this study (Fig. S4). We selected two transgenic lines (lines 1 and 2) for the investigation of flower development. Compared with WT (Fig. 2A,B), the pistils of *SlNCED1-*OE-2 flowers were deformed, with shorter and thicker styles (Fig. 2C–F). From stages 6 to 20, the WT ovary develops gradually but does not expand rapidly before pollination and fertilization (Fig. 2A,B). Compared to WT, the ovaries in flowers of the OE-2 transgenic line began to expand at stage 16 (4 DBFB), even though they were not pollinated (Fig. 2D,G). By measuring the transverse and longitudinal diameters the of the ovary before and after full bloom in the WT and transgenic *SlNCED1-OE*-2, we found that the WT ovary does not expand at 20–21 stages (0–3 days after full bloom) (Fig. 2G). The ovary begins to expand at stage 22 (on the 4 DBFB) when the petals and anthers start to fall off. By stage 23 (7 DBFB), the stigma is completely detached from the ovary. The expression of *SlNCED1* and the ABA accumulation in ovaries of the OE transgenic lines were obviously increased while they were reduced in the RNAi lines compared to the WT (Fig. 2H,I). Since the RNAi lines did not significantly change in the ovary size and the stigma morphology as the OE lines, we mainly investigated the phenotypes of OE ovaries.

### Development of the ovary in WT flowers during fruit set

**Figure 3:**
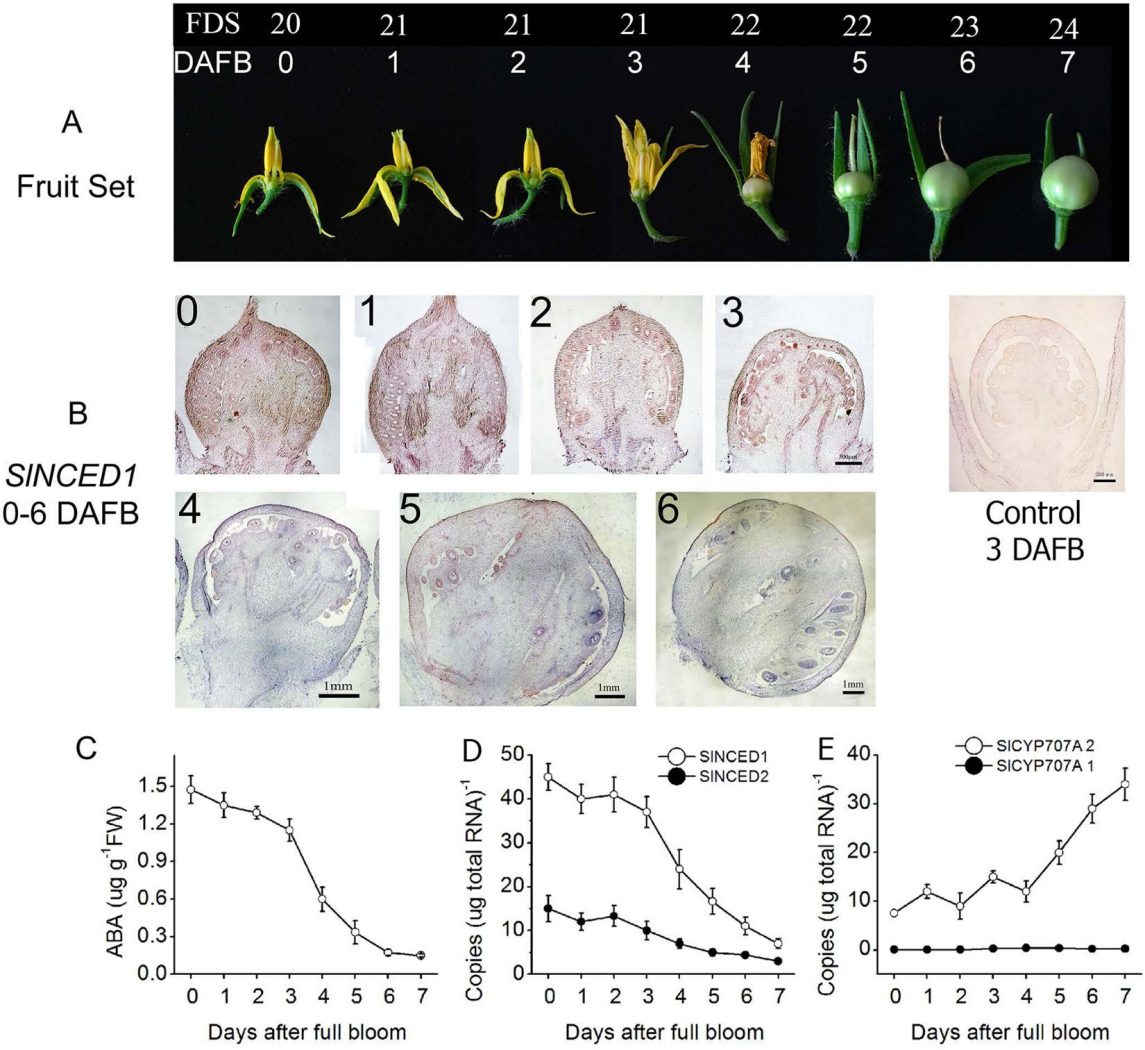
Flower morphology and young fruit development and changes in the transcriptional levels of ABA metabolism genes after full bloom (DAFB). **(A)** Morphological changes in the pistil 0–7 DAFB during fruit set. **(B)** Spatio-temporal expression of *SlNCED1* in the ovary 0–6 DAFB during fruit set. For *SlNCED1 in situ* hybridizations, the scale bar = 500 μm (0–3 DAFB), and 1 mm (4–6 DAFB). **(C)** ABA levels in the ovary during fruit set. **(D**,**E)** Changes in the transcriptional levels of ABA metabolism genes *SlNCED1/2* **(D)** and *SlCYP707A1/2* **(E)** from 0–7 DAFB. *SAND* was used as an internal control for the normalization of gene expression. Three biological replicates (n = 3) were used for each analysis. Error bars indicate the SE.

To fertilize the egg cell, pollen grains have to germinate and the pollen tubes have to grow through the stylar tissue to reach the ovule and penetrate the embryo sac. As shown in Fig. 3A, the ovary begins to expand rapidly four DAFB (days after full bloom) when the petals fall off the ovary. The stigma drops away completely from the ovary 7 DAFB. After pollination and fertilization, the ovary generally swells to become a fruit. *In situ* hybridization showed that *SlNCED1* is largely expressed in the seed, fruit peel, and vascular tissue from 0–6 DAFB (Fig. 3B). As shown in Fig. 3C, the ABA content in the ovary stays at a high level from 0 to 3 DAFB, after which it declines rapidly to near 0 at 7 DAFB following senescence and abscission of the flower petals and the expansion of the fruits (Fig. 3C). The expression of both S*lNCED1* and *SlNCED2* declines steadily from 0–7 DAFB (Fig. 3D). The expression pattern of *SlNCED2* was similar to that of *SlNCED1*, although the relative expression level was lower. To the contrary, the expression of *SlCYP707A2* was relatively low at 0–4 DAFB, but then it increased rapidly by almost 4-fold at 7 DAFB. The expression of *SlCYP707A1* was almost undetectable during fruit set (Fig. 3E). These results indicate that *SlNCED1* plays a role in ovary development and fruit set in tomato.

### Effects of *SlNCED1*-OE on ovary development during anthesis and following fruit set

**Figure 4:**
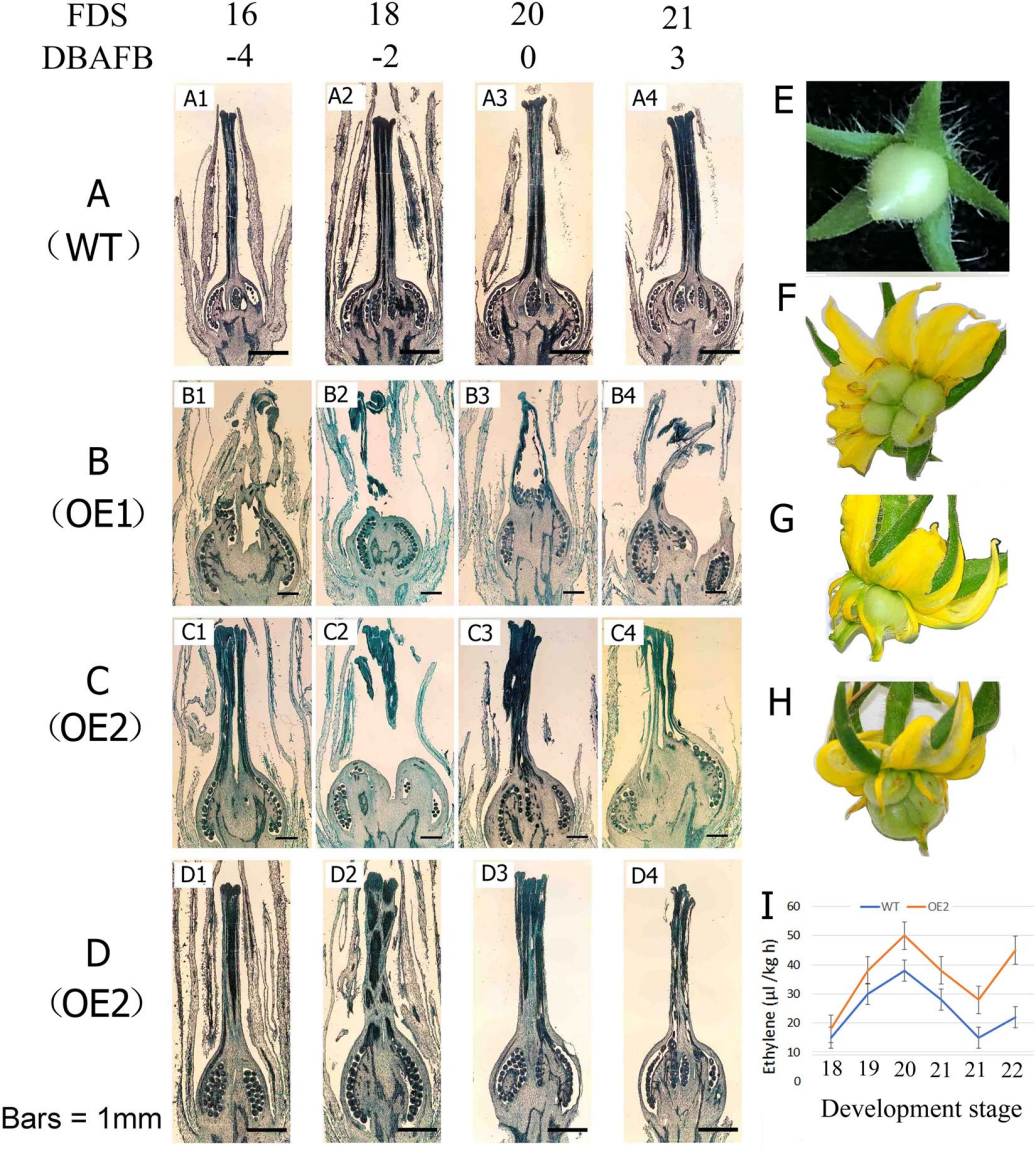
Comparison of pistil development between WT tomato (cv. ‘JianBao) and the two *SlNCED1-OE* lines before and after full bloom. Photomicrographs of pistil cross-sections from the WT and *SlNCED1-*OE-1 and -2 lines at -4, -2, 0, and 3 DAFB. **(A1–A4)** Pistils of WT flowers. **(B1–B4)** Transgenic OE-1 pistils. **(C1–C4)** Transgenic OE-2 pistils. **(D1–D4)** Cross-sections of pistils from the transgenic OE-2 line showing development of the ovaries in the absence of pollination. **(E)** WT young fruit at 5 DAFB. **(F–H)** Abnormal fruits observed in the transgenic OE-1 and OE-2 lines. **(I)** Ethylene release profile in WT and *SlNCED1-OE*-2 ovaries during anthesis (−2 to +3 DAFB).

After pollination, fertilization, and successful fruit set, the fruits begin to expand normally. We examined cross-sections of pistils from flowers of the WT cv. ‘JiaBao’ and the *SlNCED1*-OE-1 and -2 lines during the flower development stages 16–21 using a microscope. At stage 16 (four days before full bloom), the transverse diameter of WT ovary is usually 1.5 mm (Fig. 4A1). However, the ovary sizes in OE-1 and OE-2 were significantly larger than in WT at the same time (Fig. 4B1,C1). In the subsequent stages, compared to WT ovary (Fig. 4A2–A4), the ovary sizes in the two OE lines were all significantly larger (Fig. 4B2–B4,C2–C4). Among the OE lines, the ovaries in B1 to B4 showed serious malformation, and the base of style appeared to have no definite shape; in B4 and C2, the pistils were split with multiple fruits forming from a single ovary; the styles shown in C1 to C4 styles were deformed. Figure 4D shows the ovaries from the OE-2 line that developed in the absence of pollination and fruit-setting which fall off during 5 DAFB; they are the same size as in WT, but the shapes of these ovaries and the styles are very different (Fig. 4D1–D4). Most of the fruits were deformed (~70% of all fruits showed the deformity), and the deformed fruits could be divided into two categories in which (1) the ovary was malformed, or (2) the style showed deformities compared to the WT fruits (Fig. 4E–H). We also observed that the percentage of fruit set in the transgenic lines was <10%. Over 90% of the fruits were parthenocarpic and among them, 30% were regular in shape while the others were abnormal. There were no seeds in the parthenocarpic fruits because the ovaries expanded before pollination. Figure 4I shows that the ethylene release in the OE transgenic ovaries was higher than in the WT ovaries, but that the profiles were identical from stages 18–21. These results indicate that the *SlNCED1*-OE plants had enhanced ABA levels in the pistil, which led to an increase in the ovary diameter and a shortening of style and stigma, as well as infertility in the plants.

### Overexpression of *SlNCED1* affects expression of ABA-responsive genes

**Figure 5:**
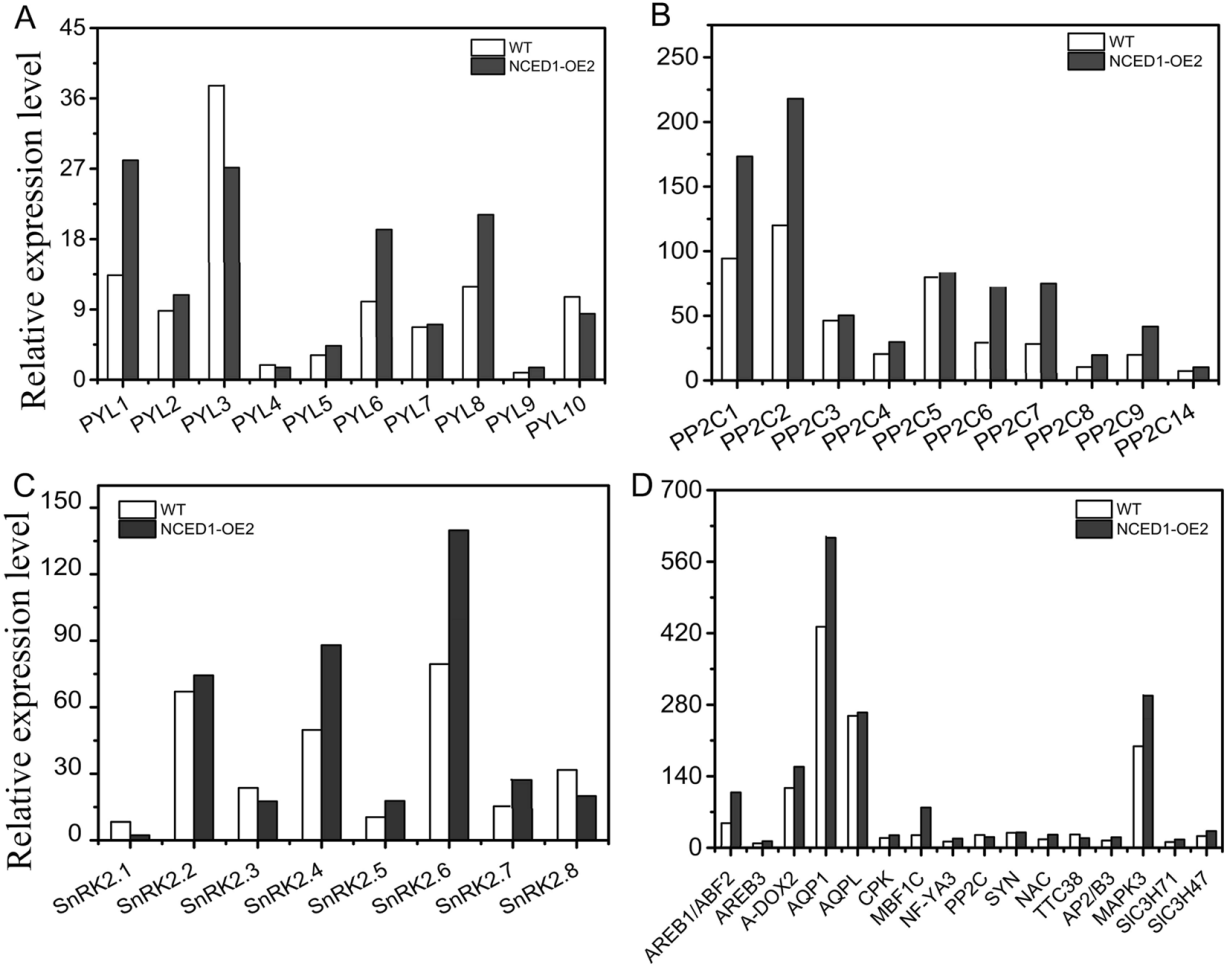
Relative expression levels of genes related to ABA signaling and transcriptional factors in WT and *SlNCED1-*OE-2 transgenic ovary. Gene expression was examined at stages 13–14 through RNA-Seq analysis.

**Figure 6:**
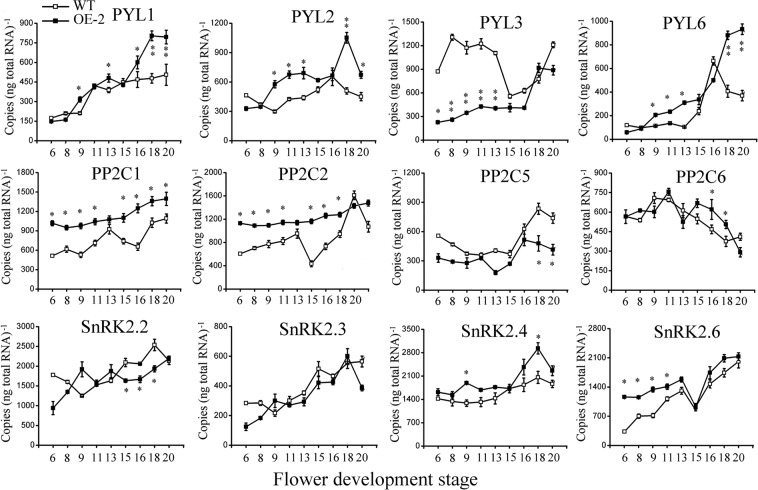
Expression patterns of genes related to ABA signaling pathway in WT and *SlNCED1-*OE-2 transgenic ovary during floral development. Gene expression was examined by qRT-PCR analysis. *SAND* was used as an internal control for the normalization of gene expression. Three biological replicates (n = 3) were used for each analysis. Error bars indicate the SE.

**Figure 7:**
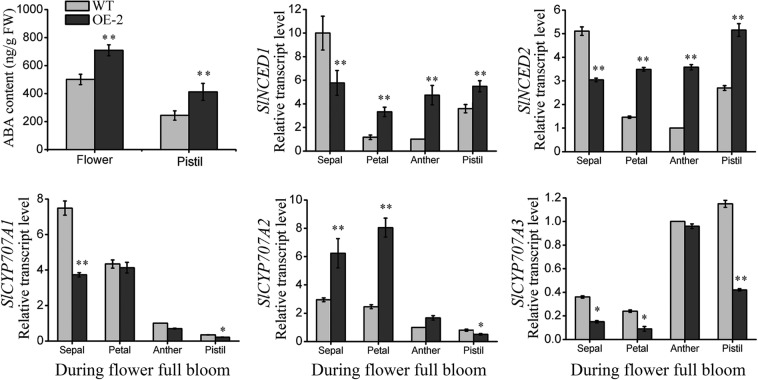
Comparison of ABA content, *SlNCEDs* and *SlCYP707As* expressions in different floral tissues between the WT and *SlNCED1-*OE-2 transgenic line at stages 13–14. Gene expression was examined by qRT-PCR analysis. *SAND* was used as an internal control for the normalization of gene expression. Three biological replicates (n = 3) were used for each analysis. Error bars indicate the SE. *P-value t-test < 0.05; **P-value t-test < 0.01.

Because two *SlNCED1*-OE lines show obvious defects in pistil development, *SlNCED1*-OE-2 was selected for further RNA-seq analysis of the pistil at developmental stages 13–14, when the embryo sac develops into a functional megaspore. The fragments per kilobase of transcript per million fragments mapped (FPKM) values of the samples were used for comparison. On the basis of a cutoff threshold of | Log2 (fold change) |>1 and p-value < 0.005, we identified 1,293 differentially expressed genes (DEGs), in which 912 were down-regulated and 381 were up-regulated, suggesting that *SlNCED1* extensively affected the ovary cell transcriptomes. RNA-Seq analyses showed that expressions of *SlPYL1/6/8* were up-regulated in OE ovary and there were no obvious changes in the expressions of other PYL family members (Fig. 5A). Among the SlPP2C genes, most of the *SlPP2Cs* were up-regulated in OE ovaries (Fig. 5B). For the *SnRK2s*, *SlSnRK2*.*4/2*.*6* were up-regulated in OE ovaries, while others showed no obvious changes (Fig. 5C). The up-regulation of only one member (SlAREB1/ABF2) of the ABA-responsive element binding factor subgroup AREB/ABF, which mediates ABA-responsive gene transcription, was observed at stages 13–14 (Fig. 5D). The expressions of *AQP1*, *MBF1C*, and *MAPK3* were up-regulated in the *SlNCED1-*OE-2 ovary at stages 13–14 (Fig. 5D). In addition, the qRT-PCR analysis showed that expressions of *SlPYL1/2/6*, *SlPP2C1/2/6* and *SlSnRK2*.*4/2*.*6* were up-regulated in OE ovaries throughout floral development (Fig. 6). Besides, the content of ABA and the expressions of *SlNCED1/2* and *SlCYP707A1/2/3* were also investigated in different floral tissues. As shown in Fig. 7, the ABA content and the *SlNCED1/2* expression were up-regulated in OE pistil and the *SlCYP707A1/2/3* expressions were lower than that in WT pistil (Fig. 7). In short, these results suggest that *SlNCED1* is involved in the regulation of ABA signaling in JiaBao tomato during ovary development.

### Pistil-specific ABA-inducible zinc finger transcription factors

**Figure 8:**
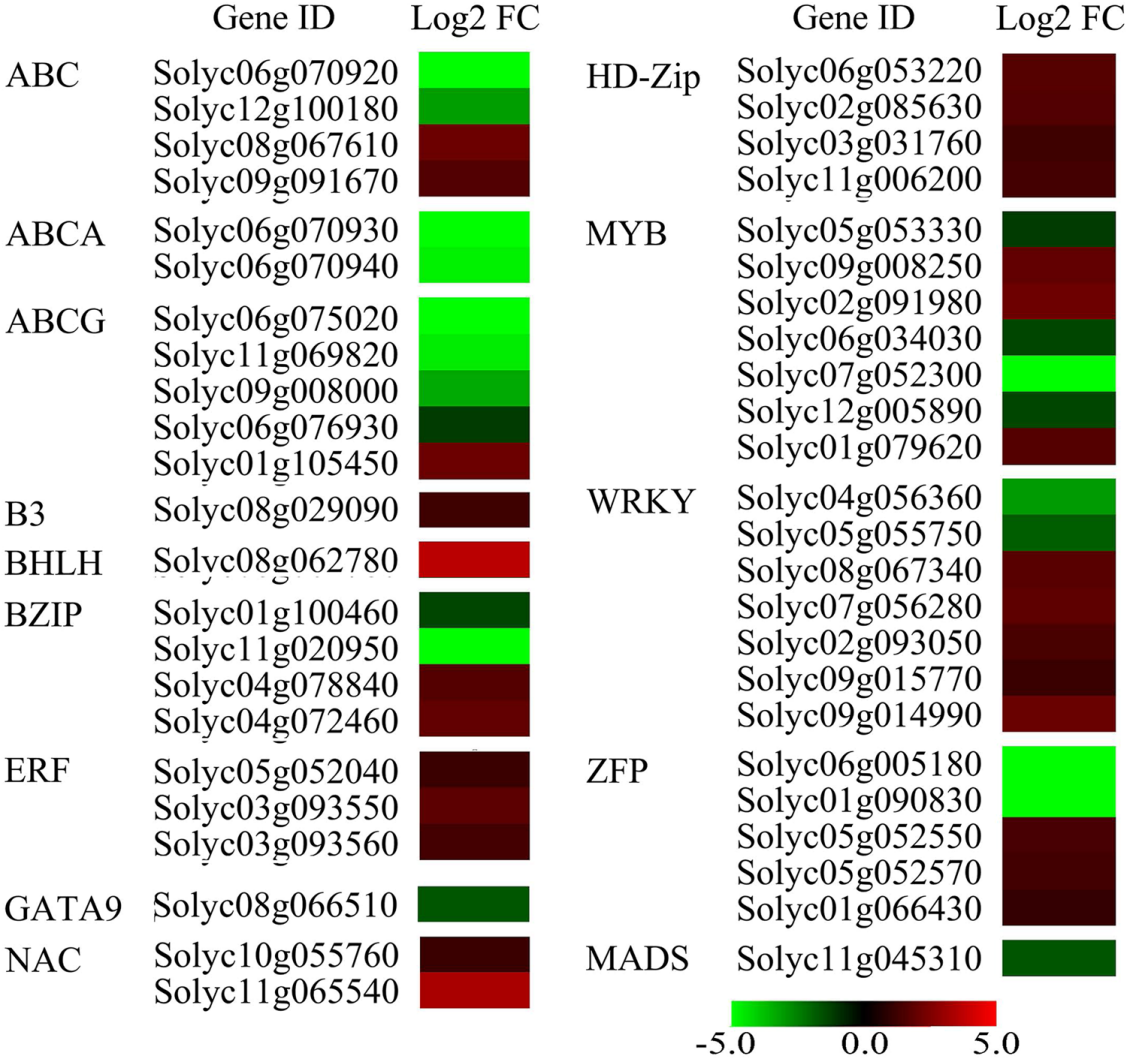
Genes encoding transcription factors involved in WT and *SlNCED1-*OE-2 pistils at stages 13–14 identified by RNA-seq analysis. Log_2_-fold change is >1 and <−1 with false detection rate (FDR) <0.005. The heat map was made using MEV4.9.0 software.

RNA-Seq analyses showed that within the group of DEGs, 47 transcription factors showed significant changes in expression (Fig. 8), suggesting that the expression of a wide range of genes is regulated in the pistil. Comparing the DEGs in the RNA-seq data between anthers^3^ and pistils at the same stage, we found six pistil-specific transcription factors, including three tandem CCCH zinc finger proteins (ZF C3H29, Solyc05g052550; ZF C3H66, Solyc05g052570; and ZF C3HC4, Solyc01g066430) and three ethylene-responsive transcription factor ERF subfamily proteins (ERF1a, Solyc05g052040; ERF, Solyc03g093550; and ERF2, Solyc03g093560), all of which were up-regulated (Fig. 8). qRT-PCR results confirmed that the expression levels of ZF *C3H66* and *ERF1a* in OE-2 pistils were up-regulated compared to WT pistils (Fig. S5, Table S3). In addition, data analysis showed that the expression of several model functional genes that encode ATP-binding cassette (ABC) transporters belonging to the conserved family of ATP-binding proteins that use ATP-derived energy to transport molecules across cell membranes^34,35^ was significantly altered in the *SlNCED1*-OE-2 pistil (Fig. 8).

### *SlNCED1*-OE alters the expression of genes related to pistil/ovary development

We further analyzed the downstream differentially expressed genes (DEGs) in the transcriptome data and the results are summarized in Supplementary Tables S4–S13. The RNA-seq analysis showed that many downstream DEGs are associated with ovary/pistil development in *SlNCED1*-mediated ovary/pistil development. Similar to the anther^3^, the expression of most of these DEGs was markedly down-regulated in the *SlNCED1*-OE-2 transgenic pistil for genes such as the receptor-like protein kinases (PRK), GTPase, and calcium ion (Ca^2+^) signaling proteins (Tables S6, S7). Carbohydrates and lipids are indispensable in pistil development, and the expression of most genes related to carbohydrate and lipid metabolism was down-regulated, indicated that the metabolism and transport of basic nutrients is defective in the *SlNCED1*-OE-2 transgenic pistils (Tables S9, S10). In addition, actin is an important cytoskeleton component that regulates cell division in pistil development. Overexpression of *SlNCED1* cause down-regulation of the expression of genes involved in the cytoskeleton, actin, and cell wall metabolism, including genes encoding actin, actin-depolymerizing factors (*SlADFs*), and formins in the pistil (Table S11). Cell wall components play an important role in pistil development, and the pistil interacts with pollen tubes during their growth; for example, β-galactosidase (BGAL) genes, cellulose synthase-like (CSL) genes, fasciclin-like arabinogalactan protein (FLA) genes, pectin lyase genes, polygalacturonase (PG) genes, pectinacetylesterase (PAE) genes, and pectin methylesterase (PME) genes encode cell wall components. Most of these genes were found to be down-regulated in the *SlNCED1*-OE-2 transgenic pistils (Table S12). In addition, many genes involved in biotic and abiotic stress responses were active in the *SlNCED1*-OE-2 pistil (Table S13).

## Discussion

### *SlNCED1* plays an important role in the regulation of ovary development

Flower development is a critical step in the plant life cycle and is controlled by complex gene regulatory networks. In order to understand whether ABA plays a role in the regulation of pistil development, we investigated the pistil/flower phenotypes and transcriptional regulation in *SlNCED1-*OE lines during flower development. During the early stages of bud differentiation (stages 4–5), *SlNCED1* is highly expressed in the pistil meristem^3^, suggesting that *SlNCED1* is involved in early tissue differentiation. Following flower development, *SlNCED1* expression is mainly restricted to the ovule, stigma, and style of the pistil during stages 6–13. This is in agreement with previous reports, in which *SlNCED1* was shown to be involved in the closest floral organs to the pistil, inside like the ovules^36^ and outside like the stamens^3,37^. Our data show that *SlNCED1* regulates stamens and pistil development at the same time, but in different roles. For example, in stages 13–15, *SlNCED1* promoted the development and maturation of pollen in the stamen^3^. Unlike in the stamen, *SlNCED1* expression and ABA content both increased in the pistil, and they were involved in at least three aspects: (1) in the ovules, they prevented pollination before pollen maturation and actively repressed ovary growth until the growth signals which originated in the newly fertilized ovules were present, (2) they promoted the absorption of sugars and nutrients in the ovary, preparing it for pollination and fertilization^38^ and (3) ABA reached higher levels in the ovary before fertilization, which might also keep the ovary tissue in a state of temporal dormancy to prevent its growth prior to pollination and fertilization. The *SlNCED1-*OE transgenic lines showed a characteristic set of developmental defects, and the floral organs developed abnormally (Fig. 4). In addition, *SlPP2C5*, which negatively regulates ABA signaling, was significantly up-regulated in the *SlNCED1*-OE-2 pistil (Table S4), suggesting that overexpression of *SlNCED1* reduced the activity of ABA signal transduction in the transgenic pistil during developmental stages 13–14. These results indicate that ABA is an essential hormone required throughout development of the ovary/pistil via ABA signaling.

### Pistil-specific *SlNCED1*-mediated zinc finger proteins

The ABA function is mainly realized through ABA signal transduction. The ABA receptor PYL and PP2C are the key components in ABA signaling, their interaction can activate the downstream signaling genes to evoke ABA responses. Our results showed that overexpression of *SlNCED1* altered the expression of ABA receptor PYLs, PP2C and SnRK2s in ABA signaling during development (Figs 4, 5). *SlNCED1-*OE affected the expression of a number of transcription factors including ZFP family. Zinc finger protein (ZFP) genes constitute a large and diverse gene family, and ZFPs have been classified into several different types, including C2H2, C2C2, C2HC, C2C2C2C2, C2HCC2C2, and CCCH, based on the number and order of the Cys (C) and His (H) residues binding the zinc ion in the secondary structure of the finger^39–41^. In this study, three pistil-specific CCCH-ZFP genes (C3H29, C3H66, and C3HC4) which are expressed only in the pistil at stages 13–14 but not in the anther, were found to be up-regulated in the *SlNCED1*-OE pistil (Fig. 8). This result indicates that these three pistil-specific CCCH-ZFP genes are involved in *SlNCED1*-mediated pistil development. Of the three tandem CCCH ZFPs, SlC3H29 and SlC3H66 share high sequence homology with *Arabidopsis* ZFP At2g40140, and SlC3HC4 is most similar to *Arabidopsis* At3g16720 (Fig. S6). *Arabidopsis thaliana* tandem CCCH ZFPs (AtTZFs) can affect plant growth and development, enhance stress tolerance, modulate ABA, GA and sugar responsive gene expression, and act as a positive regulator of ABA and a negative regulator of GA^42^. In addition, the expression of several C2H2-ZFP genes which are considered to be indispensable in the transcriptional regulation of floral organ morphogenesis and pistil development^23^, were down-regulated in the *SlNCED1*-OE pistil during floral development, providing evidence of their involvement in ABA-mediated pistil development. The RNA-seq analysis showed that several ABC model function genes were down-regulated in the *SlNCED1*-OE-2 pistil. From this result we can speculate that these ABC model function genes are associated with the alteration of ZFP gene expression, because the ZFPs can directly or indirectly regulate the ABC model function genes in floral organ development by affecting hormonal signaling in cell proliferation and division^35,42^.

ABA is involved in many aspects of plant growth and development and plays important roles in many cellular functions, including transcriptional regulation in pistil development and the associated ZFP TF regulation, which suggests a mechanism of pistil development by ABA and ZFP TF co-regulation. Despite the effects of *SlNCED1*-OE on ovary development found in our study, the major issues remain unclear. For instance, although *SlNCED1* has been shown by *SlNCED1*-OE in transgenic plants to participate in ovary development, limited information is available on the exact process by which ABA regulates the pistil and about the regulatory network. In addition to ZFPs, numerous TFs are involved in flower development. Thus, future research should explore the exact function of ABA in the regulation of flower development, and the interaction between ABA and ZFPs or other TFs. Also, we found that three ethylene response factor (ERF) genes that belong to the ethylene-responsive transcription factor ERF subfamily in different clades and are homologs of *Arabidopsis* At5g47230, were up-regulated in the *SlNCED1*-OE-2 pistil (Figs 8, S7). The ERFs are considered to be integrators of hormonal pathways and are directly responsible for the transcriptional regulation of several jasmonate (JA)/ethylene - responsive defence genes^43–45^.

### ABA is involved in the regulation of fruit set in tomato

Anthesis is a crucial developmental phase in which ovary growth is actively repressed until the signals that promote ovary growth originate in the newly fertilized ovules^27,46^. The hormonal balance between ABA, auxin, gibberellin, and ethylene in the ovary determines the maintenance of cellular homeostasis and the ovary-fruit transition. The roles of auxin and gibberellin in the ovary during fruit set are well studied^47–49^; however, the function of ABA in this process is unclear at present due to a scarcity of molecular evidence. It has been reported that the auxin burst induced by pollination is the first positive signal in fruit set, and the functional interaction between Aux/IAA and ARF proteins is one of the most well-characterized features of this mechanism^36^. With respect to GA, pollination activates GA metabolism/response by inducing the transcription of specific genes, such as those involved in GA biosynthesis^47,48^.

ABA has been suggested as a repressor of fruit set^47^ in contrasting role with IAA and GA that are promoting hormones. However, our data shows that increased ABA content caused by *SlNCED1*-OE allowed fruit set without fertilization, leading to parthenocarpic fruit development (PFD) in tomato. ABA plays an important roles in fruit set through transcriptional regulation in the hormone balance and in the associated transcription factor (TF) such as ZFP, ERF, and MYB regulators (Table S5; Fig. 8), which suggests a mechanism of fruit set by ABA and associated TF co-regulation in addition to IAA and GA. In this work, the ABA levels and the *SlNCED1* expressions are maintained at relatively higher levels several days before and after full bloom. Meanwhile, *SlNCED1*-OE/RNAi not only significantly affected the pollen and ovary development, but also worsened fruit set and the young fruit growth (Figs 2–4). These results suggested that the right amount of ABA played a positive role in the development of male and female gametes and in the subsequent pollination, fertilization and fruit set. RNA-seq analysis showed that *SlNCED1*-OE/RNAi significantly altered the expression of genes related to ethylene, indole-3-acetic acid (IAA or auxin), cytokinin (CTK), gibberellins (GAs), jasmonic acid (JA), and salicylic acid (SA) (Table S4), suggested that ABA maintained the hormonal balance before fruit set through transcriptional regulation of the relevant genes. Then, during fruit set, the ABA content and *SlNCED1/2* expression in ovary were higher than that in anther and petal while the *SlCYP707As* expressions were the opposite of *SlNCEDs*, suggested that ABA was given priority to the ovary in fruit setting process which further explained the indispensability of ABA during fruit set.

In our study, WT tomato plants (cv. ‘JiaBao’) accumulated higher levels of ABA and had lower IAA/GA levels in the ovary from six days before flowering to 1–3 days after full bloom (Fig. S8). However, in transgenic *SlNCED1-OE*-1 and -2 plants, in the ovary, the ABA content was higher than WT. Increased ABA levels due to *SlNCED1* overexpression may induce early IAA/GA accumulation before fruit set, which caused the ovaries to enlarge in the absence of fertilization, leading to parthenocarpic fruit development (PFD) in tomato. It is worth noting that we also observed abnormal ovaries and parthenocarpic fruits in the *SlNCED1*-RNAi lines similar to the *SlNCED1*-OE lines, but the phenotypes were not as severe. A balance among IAA, GA, ABA and ethylene levels is suggested as a mechanism underlying the ABA-only or IAA/GA-only-induced PFD before and after full bloom. We found that the genes that are putatively involved in the control of ovary growth before pollination^36^ showed down-regulated expression before and after full bloom (pollination) in the *SlNCED1-OE* transgenic ovary (Tables S4–S13). These results show that ABA may be involved in the regulation of hormone levels in the tomato ovary during fruit set and the exact effect of ABA on IAA and GA levels needs further experimental evidence.

## Conclusion

Based on the results of our study, we can conclude that (i) *SlNCED1* plays an important role in the regulation of pistil development and fruit set via the ABA signaling pathway, (ii) ABA signaling activates the expression of pistil-specific transcription factor CCCH-ZFPs, which regulate the expression of related downstream genes, and (iii) increased ABA levels caused by *SlNCED1-*OE in the ovary result in an imbalance in the hormone levels, thereby inducing parthenocarpic fruit development.

## Supplementary information

Supplementary information
