# Supplementary material for: Functional analysis of SlNCED1 in pistil development and fruit set in tomato (Solanum lycopersicum L.)
Source: Sci Rep. 2019 Nov 15;9:16943. doi: 10.1038/s41598-019-52948-2 (PMC6858371; doi:10.1038/s41598-019-52948-2)
Supplement: Supplementary file 1 — Supplementary information [file 41598_2019_52948_MOESM1_ESM.pdf]

Functional analysis of *SlNCED1* in pistil development and fruit set in tomato (*Solanum lycopersicum* L.)

Wenbin Kai†, Ying Fu†, Juan Wang, Bin Liang, Qian Li*, and Ping Leng*

Supplementary data

Figure legends

Figure S1. Comparison of growth and development between WT, *SlNCED*-RNAi, and *SlNCED*-OE transgenic tomato plants.

Figure S2. Expression of ABA signaling genes (*PYL*, *PP2C*, and *SnRK2* family members) in the tomato ovary during floral development.

Figure S3. Expression of genes related to ethylene synthesis and signaling in the tomato ovary during floral development.

Figure S4. Comparison of *SlNCED1* expression levels in WT and *SlNCED*-OE transgenic tomato ovaries. *SAND* was employed as an internal control. Three biological replicates ($n = 3$) were used for each analysis. Error bars are SE. **P* value *t* test < 0.05; ***P* value *t* test < 0.01

Figure S5. Relative expression of *ERF* and *ZFP* transcription factor genes in pistils of WT and *SlNCED*-OE transgenic tomato plants during development.

Figure S6. Phylogenetic relationships of zinc finger protein family members from *Arabidopsis thaliana* and tomato *(Solanum lycopersicum*).

The black circles and the while triangle indicate tomato SlZFP and *Arabidopsis* AtZFP, respectively. All protein complete sequence can query to obtain using by gene ID, as below: Arabidopsis database: https://www.arabidopsis.org/
Tomato database: https://solgenomics.net/

Figure S7. Phylogenetic relationships of ethylene response factor (ERF) family proteins from *Arabidopsis thaliana* and tomato *(Solanum lycopersicum).*

The black circles and the while triangle indicate tomato SlERF and *Arabidopsis* AtERF, respectively.

All protein complete sequence can query to obtain using by gene ID, as below:

Arabidopsis database: https://www.arabidopsis.org/

Tomato database: https://solgenomics.net/

Figure S8. Comparisons of changes in hormone levels and gene expression in ovaries of WT and *SlNCED1-OE*-2 flowers from 6 days before to 6 days after full bloom. **(A)** ABA content. **(B)** Expression of *SlNCED1*. **(C)** Auxin content. **(D)** GA content. Transcript levels were normalized to the expression of the house-keeping gene *SAND*. Three biological replicates (n = 3) were used for each analysis. *P value t test <0.05; **P value t test <0.01.

Supplemental Table 1. Specific oligonucleotide primers used for amplification of genes in this study.

Name	Oligonucleotides
35S-prom-f (SacI)	5'-GAGCTCAGATTAGCCTTTTCAATTTCAGAAA-3'
35S-Prom-r(XbaI)	5'-TCTAGACGTGTTCTCTCCAAATGAAATGA-3'
Nos-f (Hind 111)	5'-GAATTTCCCCGATCGTTCAAACATTTGGC -3'
Nos-r (Sal 1)	5'-CCGATCTAGTAACATAGATGACACCGCGC-3'
NCED1-sense-f (EcoR 1)	5'-TGGGTCGCCCTGTTTTCCCTAAAGCCATT-3'
NCED1-sense-r (Hind 111)	5'-TCATGCATCATTGTTGGGTCTTCAACTGG -3'
NCED1-anti-f (BamH 1)	5'-TGGGTCGCCCTGTTTTCCCTAAAGCCATT-3'
NCED1-anti-r (Xba 1)	5'-TCATGCATCATTGTTGGGTCTTCAACTGG -3'
NCED1-OE-f (BamH 1)	5’-ATGGCAACTACTACTTCACATGCCAC-3’
NCED1-OE-r (Hind 111)	5’-TCATGCCTGATTTGCCAAATCATTGGCG-3’
GUS-f (Pst 1)	5'-TGGTCAGTCCCTTATGTTACGTCCTGTAG -3'
GUS-r (EcoR I)	5'-GGTAGCAATTCCCGAGGCTGTAGCCGACG-3'

Supplemental Table 2. Oligonucleotide primers used for quantitative real-time PCR analysis.

Gene ID	Target gene	Forward primer(5'-3')	Reverse primer(5'-3')
Solyc03g115810	*SlSAND*	TTGCTTGGAGGAACAGACG	GCAAACAGAACCCCTGAATC
Solyc07g056570	*SlNCED1*	AGGCAACAGTGAAACTTCCATCAAG	TCCATTAAAGAGGATATTACCGGGGAC
Solyc08g016720	*SlNCED2*	TGGTTTTCATGGGACATTCATTAGC	ATCTCCCTTCTCAACTCCCTATTCC
Solyc04g078900	*SlCYP707A1*	CCCAGAGTTCTTTCCTGATCCACAA	GAATGCCACTACCAGATCCTACCAC
Solyc08g005610	*SlCYP707A2*	TCGAAAAAGGATACAATTCGATGCC	CTGCAATTTGTTCGTCAGTGAGTCC
Solyc08g075320	*SlCYP707A3*	CTCATGTCCAGGCAATGAGTTAGCC	GGAGCGAAGCTAGAATGAGAATCACC
Solyc08g082180	*SlPYL1*	TATGCCGGTCCTTCTCGATCTCA	AAGCTCAATTCACGCACAGCCAC
Solyc12g055990	*SlPYL2*	TTAGCTGCTGGAGCGTACATGGA	ACGCCTTTGCCACAGTAGGGTAG
Solyc01g095700	*SlPYL3*	ATCACAGGCTAAAGAACTATTCTTCGG	ACCAGTTCACACTGACGGGTAGAAC
Solyc03g095780	*SlPYL4*	TCACTTACATTACCACACCCACGCT	TTACCTGAACCTCCCTTAATGTGCC
Solyc08g076960	*SlPYL5*	TGAGGAATTACCGTTCAGTTACGACG	CTCGCATCACGACTATTAACACTACCA
Solyc06g050500	*SlPYL6*	AGCGGTTAAACAACCGCAAC	TTATCGAATCGGCGGACGAG
Solyc10g085310	*SlPYL7*	TGGATCGATCAACTTGCTCTAACGC	TAAAGGTGCAACGAAACAATCATGC
Solyc06g061180	*SlPYL8*	CTAGTGTACATGGATTCGAACGCCA	AGATCTCACCGTTACCACCAAGAGC
Solyc09g015380	*SlPYL9*	ACCACCAGCACCTCCAGTAAACAA	GACGAAGTGTTTGTATGCTTGAGGG
Solyc03g007310	*SlPYL10*	CACCCAGAGGTGATTGATGGAAGA	TCAATGGGTTCTGTCCTGTCTTGC
Solyc10g076410	*SlPYL11*	CGAAGAGCGCCACGTTATTAGTTT	TTAAATTCTCCGCGATCTGTTTCAG
Solyc03g096670	*SlPP2C1*	AGAATAAAGAAACCGAAACGAACGC	ATCCTGAAGAAACGACGGGTAGATC
Solyc05g052980	*SlPP2C2*	CAGTGATGGATTATGGGACGTGGTA	CCTAGCCAAGGCTAATTTCGTCAA
Solyc06g076400	*SlPP2C3*	TGGCAGAGTAATCTACTGGGACGG	TGAGACTACGTCCCATAATCCGTCA
Solyc07g040990	*SlPP2C4*	GGTACTTTGAGTAGGGAAAGGGGTGA	GAAATACGAGGATGGTTTAGTGCGTTA
Solyc03g121880	*SlPP2C5*	GTGTATTTGGCGTTCTTGCAATGTC	CAGGCAGAGGGTTAGTCCCGTTC
Solyc12g096020	*SlPP2C6*	TGGTAAGACTGGGGTTTTGGTTGA	TCTAGGAACAGTTGCGAATGCGTC
Solyc08g062650	*SlPP2C7*	AAGTCAATGGCTGACCATGCTCC	TGGAATCACTCACCACAGACAGGG
Solyc05g056550	*SlSnRK2.1*	TGAGCGAGGGCGTAAGATTG	ATCGAGCTTCGGGTTCACTG
Solyc01g103940	*SlSnRK2.2*	CTCCAGTTTCCCGTTCAGTCTCAG	CTGCGAGCAAGCAAGATAATTTACG
Solyc01g108280	*SlSnRK2.3*	ATCATTACCTCACTGGAAGCTTGGAC	AAACAGTGGATACCAAAAGATCGCC
Solyc02g090390	*SlSnRK2.4*	TTCCAGCGGACTTAATAGACGATAGG	TCACAAGCTTGGCATGCACTCAC
Solyc08g077780	*SlSnRK2.6*	CAGCAATGACTTTGCTGTTTATGTCTG	CGTGAATCCGAATTAGACGGATACC
Solyc04g074500	*SlSnRK2.7*	TCCAGAAATTAAAATGCATCCCTGG	AATGTGTTCCAACCTGCAGAGGAAC
Solyc04g012160	*SlSnRK2.8*	CTAACGCAGGATGTTTTGTGCACC	AATATGGAGTCCTTGGTGTCATACATGA
Solyc08g081550	*SlACS1A*	GCATCAATGTTGTCTGATGAAGTATTCA	GCAATGTTGTTAAGTCCCTTTGGC
Solyc01g095080	*SlACS2*	CTACGCAGCCACTGTCTTTGAC	TGATTCCGACTCTAAATCCTGGTAA
Solyc05g050010	*SlACS4*	TTGCGACGAAATATATGCTGCT	CACTCGAAATCCTGGAAAACCT
Solyc07g049530	*SlACO1*	ACTATCCACCATGTCCTAAGCCCG	TCTGTTTGTGCAATTACTCTGTGCAGC
Solyc09g075440	*SlETR3*	AAGGGAACCACTGTCACGTTTGTAG	TTAATGTTCTTTGTCACACCAATGTCC
Solyc10g083610	*SlCTR1*	ACATTTGGATTATGTCAGGCTTGCA	TTGCTCAAACAATGGTTCAAAGAGG
Solyc09g007870	*SlEIN2*	AAGTTCTTGGTGATGTCAGTTCCCC	TCTACTATGCCCTGAAGACGGTTGAG

Solyc09g075420	*SlERF2*	GAAAAGGGCTCCTCAGAGAGCATAT	TTTCTTCTGCCAATTCAAGTGACGA
Solyc03g095900	*SlE8*	AACAGTATTACACTCGAGATACTGCGA	TGAGATAACTACGATCGAGACCGAG
Solyc02g081120	*LeT6/TKn2*	CCATCGTCTCTTGACTGCTTATCT	AGGATCTTCTCCAATGATTCCACC
Solyc11g005740	*SlBPEp*	CAAAGTAGTAAACCTGAGCCACCT	TTGCAGAATTTTCATCCTCTCGCT
Solyc05g012050	*SlCRCb*	GCACCTTTTGTTGTAAAACCTCCT	AGCTTCTCTATGTGGTATCTCTGG
Solyc04g081000	*SlDEF*	TAAGTCCCTCTATCACGACCAAAC	TCCTATTCACATCCTTTAGCTTCC
Solyc11g013310	*SlLAX3*	AATGGACAGTTGAGGGAGTG	ATTAGAGTGGGTGAGAAGTGC
Solyc03g120980	*SlPEN3*	TTTATAGAGAAAGGGCTGCTGG	TGGTTCGGTGTGATGGAAAC
Solyc10g080890	*SlGAN*	ATTTGTCAGAGGAGGAATGGGAGA	CGATTAAGACCAGCGGTAGAAGAG
Solyc09g074270	*SlGID1*	TACAAACCTGCCCCGAAAAATGAG	ACCGTGGAAGAAGATAATGACAGG
Solyc01g058250	*SlGA3ox*	CTTACCACGCACTGGGGTTAGC	CAATGAGGGCATCGTTGGAAA
Solyc06g035530	*SlGA2ox 2*	TTTCCATATTCTACCCTACAAG	TCATCGCATTACAATACTCTT

Supplemental Table 3. Oligonucleotide primers used for measuring the expression of transcription factor genes in the tomato pistil by qRT-PCR.

Gene names	Gene ID		Primer sequences (5' → 3')
SlERF1a	Solyc05g052040	Forward	ATAATCTCCATTTTCACTCTTCATTTTCCA
		Reverse	AAGATTTGAACTTTGAGGTGTATTGATGAA
SlERF	Solyc03g093550	Forward	TTGCTTTCATGGAATATTACTGCTCTAAAT
		Reverse	CTCTGTCTAACTCCCCTGTAATGCTTCT
SlERF2	Solyc03g093560	Forward	TTTGATGAATCTCTGGACCAGACTTGT
		Reverse	CCTCTGTCTAACTCCCCTGTAATGCTT
SlC3H29	Solyc05g052550	Forward	GTGCTGTTCCCAATGGTGTTGC
		Reverse	GTGTGATTGTTGATCTTGTTGGAGCA
SlC3H66	Solyc05g052570	Forward	TTGAGAAAGGTTGTGCTGTTGATGAGT
		Reverse	CACAGTGAAGAGCAGTTGCACCATC
SlC3HC4	Solyc01g066430	Forward	GATTCATGATCCATTGATTGATTCCACA
		Reverse	AACCGCCTGTTCCAAACCCCG

Supplemental table 4. The differentially expressed genes involved in hormone metabolism and signaling in the pistil of WT and *SlNCED1*-OE-2 at stage 13-14.

Gene ID	RPKM WT	RPKM OE 2	Log2.Fold Change	p.value	Description
**Abscisic acid (ABA)**					
Solyc07g056570	77.33	488.00	2.66	1.49E-43	SlNCED1
Solyc04g078900	13.10	56.52	2.11	5.53E-06	Cytochrome P450 (CYP707A1)
Solyc08g005610	221.64	69.02	-1.68	4.61E-12	Cytochrome P450 (CYP707A2)
Solyc12g056600	7.05	30.91	2.13	1.37E-10	ABA2
Solyc12g045030	92.19	310.87	1.75	6.80E-33	ABA2
Solyc06g071070	4.41	10.98	1.32	4.32E-03	ABA2
Solyc01g009240	1098.91	41.95	-4.71	6.14E-116	Beta-glucosidase
Solyc01g079570	18.18	49.65	1.45	7.27E-09	Beta-glucosidase
Solyc11g071650	8.44	65.06	2.95	5.15E-09	Beta-glucosidase
Solyc01g010390	117.24	658.26	2.49	5.55E-45	Beta-glucosidase
Solyc01g080370	15.33	2.93	-2.39	2.64E-04	Glycosyltransferase (GT)
Solyc01g094390	20.26	0.59	-5.11	3.48E-03	Glycosyltransferase
Solyc03g025780	128.58	57.80	-1.15	1.97E-07	Glycosyltransferase
Solyc06g008910	27.77	0.70	-5.30	2.25E-08	Glycosyltransferase
Solyc09g074080	17.21	0.40	-5.44	7.35E-09	Glycosyltransferase
Solyc06g075560	37.70	0.23	-7.37	1.32E-07	Glycosyltransferase
Solyc05g046110	94.09	0.92	-6.67	1.32E-23	Glycosyltransferase
Solyc11g065390	29.13	0.04	-9.61	1.55E-05	Glycosyltransferase
Solyc11g065400	58.92	1.78	-5.05	1.09E-13	Glycosyltransferase
Solyc11g072700	25.65	0.85	-4.91	3.63E-05	Glycosyltransferase
Solyc04g016230	1.48	6.47	2.13	1.45E-03	Glycosyltransferase
Solyc04g008310	10.38	22.70	1.13	1.17E-08	Glycosyltransferase
Solyc02g067690	10.98	26.89	1.29	5.99E-07	Glycosyltransferase
Solyc02g070020	2.01	9.31	2.21	9.32E-07	UDP-glucosyltransferase (UGT)
Solyc12g042600	14.38	48.30	1.75	6.98E-16	UDP-glucosyltransferase
Solyc06g062330	5.15	18.83	1.87	5.64E-08	UDP-glucosyltransferase
Solyc10g008860	10.58	0.07	-7.31	1.06E-05	UDP-glucosyltransferase
Solyc02g085660	33.64	8.56	-1.98	1.06E-17	UDP-glucosyltransferase
Solyc09g008060	109.80	52.79	-1.06	3.47E-27	UDP-glucosyltransferase
Solyc07g006720	72.85	3.89	-4.23	2.77E-25	UDP-glucosyltransferase
Solyc12g057060	135.17	54.35	-1.31	6.35E-26	UDP-glucosyltransferase
Solyc03g121880	123.4	497.64	1.98	1.3E-102	Phosphatase 2C family （PP2C5）
Solyc10g085370	57.69	3.15	-4.19	5.44E-08	Phosphatase 2C family protein
Solyc10g076320	140.71	8.50	-4.05	9.13E-18	Phosphatase 2C family protein
Solyc02g082990	1961.49	17.93	-6.77	1.97E-134	ABA-inducible protein-like protein
Solyc02g065640	1036.61	4.24	-7.93	2.50E-47	ABA-inducible protein-like protein
**Ethylene (ETH)**					
Solyc02g071430	305.27	104.18	-1.55	3.06E-32	ACS

Solyc06g060070	25.06	0.52	-5.58	5.13E-04	ACS
Solyc02g071440	32.57	8.74	-1.90	2.54E-04	ACS
Solyc06g068270	12.85	36.52	1.51	5.08E-05	ACS
Solyc12g006380	9.40	23.23	1.31	4.54E-03	ACS
Solyc04g009860	41.67	122.26	1.55	5.94E-18	ACS
Solyc09g089610	36.84	10.49	-1.81	1.22E-17	Ethylene receptor (ETR)
Solyc10g076450	79.74	19.19	-2.05	2.88E-21	Ethylene-overproduction protein (ETO)
Solyc08g078190	8.91	25.23	1.50	2.29E-03	Ethylene receptor factor (ERF)
Solyc03g093550	11.02	40.30	1.87	1.63E-03	ERF
Solyc06g082590	17.79	39.96	1.17	3.96E-05	ERF
Solyc06g066540	2.04	4.84	1.25	2.88E-03	ERF
Solyc09g059510	32.60	88.31	1.44	1.53E-05	ERF
Solyc05g052040	40.85	93.49	1.19	2.59E-06	ERF
Solyc03g093560	39.62	101.17	1.35	2.05E-07	ERF
Solyc05g052040	40.85	93.49	1.19	2.59E-06	SlERF1a
Solyc03g093550	13.11	45.97	1.81	1.38E-05	SlERF
Solyc03g093560	39.62	101.17	1.35	2.05E-07	SlERF2
**Auxin (IAA)**					
Solyc04g007690	15.40	39.87	1.37	7.03E-06	Auxin efflux carrier (AEC)
Solyc12g009280	88.50	1.23	-6.17	7.19E-11	Auxin-induced protein-like (AIP)
Solyc09g010230	24.45	55.94	1.19	1.31E-05	Auxin response factor (ARF)
Solyc08g082630	18.59	77.65	2.06	1.71E-06	Auxin response factor (ARF)
Solyc05g025920	56.65	3.18	-4.16	8.35E-11	Auxin responsive SAUR protein (ARSP)
Solyc01g099840	144.04	300.22	1.06	5.81E-22	Auxin-repressed protein (ARP)
Solyc03g006360	333.26	166.31	-1.00	9.60E-21	Auxin-repressed protein (ARP)
Solyc06g063060	103.60	39.64	-1.39	7.30E-11	Auxin-repressed protein (ARP)
Solyc12g005310	515.69	100.19	-2.36	4.77E-120	Auxin-responsive GH3-like (ARGL)
**Cytokinin (CTK)**					
Solyc04g080820	12.58	47.51	1.92	5.61E-09	Cytokinin oxidase/dehydrogenase (CKX)
Solyc01g098400	68.41	146.08	1.09	1.15E-03	Histidine phosphotransfer protein (AHP)
**Gibberellin (GA）**					
Solyc07g061730	3.70	26.03	2.81	3.03E-07	Gibberellin 2-oxidase (GA2ox)
Solyc02g038740	431.71	112.48	-1.94	1.38E-67	HMGR
**Jasmonic acid (JA)**					
Solyc10g061840	23.24	0.15	-7.29	1.93E-03	Carboxyl methyltransferase
Solyc01g080970	14.22	2.63	-2.44	4.06E-03	Carboxyl methyltransferase
Solyc02g065280	115.59	44.12	-1.39	1.28E-09	Methyl jasmonate esterase
**Salicylic acid (SA)**					
Solyc09g091550	18.17	44.82	1.30	2.74E-06	SAM
Solyc01g005360	147.12	0.97	-7.24	3.72E-21	SMAT
Solyc08g006330	6.03	15.72	1.38	2.64E-03	Salicylic acid glucosyltransferase

Supplemental table 5. The differentially expressed genes involved in transcriptional factors in the pistil of WT and *SlNCED1*-OE-2 at stage 13-14.

Gene ID	RPKM WT	RPKM OE 2	Log2.Fold Change	p.value	Description
**ABC**					
Solyc06g070920	354.17	2.65	-7.06	9.41E-10	ATP-binding cassette transporter 17
Solyc12g100180	322.37	37.46	-3.11	9.71E-35	ATP-binding cassette transporter
Solyc08g067610	14.92	64.00	2.10	5.06E-06	ATP-binding cassette transporter
Solyc09g091670	14.02	42.71	1.61	5.37E-04	ATP-binding cassette transporter
**ABCA**					
Solyc06g070930	85.17	0.55	-7.26	7.35E-06	ABC transporter A family member 7
Solyc06g070940	235.47	8.98	-4.71	6.92E-23	ABC transporter sub-family A member 14
**ABCG**					
Solyc06g075020	65.25	1.90	-5.10	3.74E-08	ABC transporter G family member 28
Solyc11g069820	127.02	5.15	-4.62	1.20E-14	ABC transporter G family member 28
Solyc09g008000	296.34	29.70	-3.32	7.51E-36	ABC transporter G family member 28
Solyc06g076930	1333.00	594.80	-1.16	3.63E-58	ABC transporter G family member 31
Solyc01g105450	65.80	283.48	2.11	1.00E-35	ABC transporter G family member 11
**B3**					
Solyc08g029090	14.67	35.36	1.27	3.20E-03	B3 domain-containing protein (B3)
**bZIP**					
Solyc01g100460	60.20	23.22	-1.37	1.55E-24	Basic region/leucine zipper motif
Solyc11g020950	428.61	8.05	-5.73	4.26E-39	Basic region/leucine zipper motif
Solyc04g078840	72.65	231.78	1.67	2.36E-31	Basic region/leucine zipper motif
Solyc04g072460	47.70	182.25	1.93	3.33E-14	Basic region/leucine zipper motif
**HD-Zip**					
Solyc06g053220	6.26	20.48	1.71	4.04E-05	Homeobox leucine zipper protein
Solyc11g006200	18.32	47.13	1.36	9.14E-04	Homeobox transcription factor
Solyc03g031760	24.20	58.23	1.27	4.45E-04	Homeobox-leucine zipper protein ATHB-14
Solyc02g085630	147.96	451.45	1.61	1.30E-49	Homeobox-leucine zipper-like protein
**ZFP**					
Solyc05g052550	98.88	261.22	1.401515	4.26E-19	Zinc finger SlC3H29
Solyc05g052570	221.01	555.89	1.330688	5.95E-35	Zinc finger SlC3H66
Solyc01g066430	72.56	150.02	1.047908	4.03E-07	Zinc finger SlC3HC4
Solyc06g005180	31.55	0.73	-5.41741	2.97E-18	Zinc finger C2H2-type
Solyc01g090830	3.933	0.06	-5.87238	0.001023	Zinc finger C2G2-type
**bHLH**					
Solyc08g062780	2.37	30.82	3.70	3.35E-05	Basic helix-loop-helix
**MADS-box**					
Solyc11g045310	304.39	95.46	-1.67	8.50E-15	MADS box protein
**MYB**					
Solyc05g053330	67.75	30.41	-1.16	1.10E-06	myeloblastosis
Solyc09g008250	2.08	8.01	1.94	1.70E-03	myeloblastosis

Solyc02g091980	16.40	73.83	2.17	6.11E-12	myeloblastosis
Solyc06g034030	162.73	62.19	-1.39	3.03E-09	myeloblastosis
Solyc07g052300	48.24	0.63	-6.25	3.43E-12	Myb-related transcription factor
Solyc12g005890	102.12	38.69	-1.40	7.78E-09	Myb-related transcription factor
Solyc01g079620	16.09	50.67	1.66	3.31E-06	Myb 12 transcription factor
**NAC**					
Solyc10g055760	31.93	70.19	1.14	1.22E-03	NAC domain protein
Solyc11g065540	2.26	22.88	3.34	4.28E-05	NAC domain transcription factor
**WRKY**					
Solyc04g056360	247.69	30.49	-3.02	1.32E-51	WRKY transcription factor 78
Solyc05g055750	683.12	190.69	-1.84	1.75E-79	WRKY transcription factor-a
Solyc08g067340	27.83	94.09	1.76	6.91E-11	WRKY transcription factor
Solyc07g056280	7.06	25.59	1.86	1.08E-05	WRKY transcription factor 16
Solyc02g093050	50.06	138.39	1.47	3.40E-14	WRKY transcription factor 26
Solyc09g015770	172.61	380.31	1.14	4.09E-32	WRKY transcription factor 6
Solyc09g014990	12.56	52.96	2.08	1.48E-07	WRKY transcription factor 6
**GATA**					
Solyc08g066510	24.12	7.40	-1.70	7.05E-05	GATA transcription factor 9
**ERF**					
Solyc05g052040	40.85443	93.49994	1.194473	2.59E-06	Ethylene responsive transcription factor 1a
Solyc03g093550	13.11237	45.97764	1.810004	1.38E-05	Ethylene responsive transcription factor
Solyc03g093560	39.6285	101.1719	1.352198	2.05E-07	Ethylene responsive transcription factor 2

Supplemental Table 6. The differentially expressed genes involved in receptor-like kinase signaling in the pistil of WT and *SlNCED1*-OE-2 at stage 13-14.

Gene ID	RPKM WT	RPKM OE 2	Log2.Fold Change	p.value	Description
**RLK**					
Solyc12g009190	69.79	1.17	-5.90	2.06E-29	Receptor like kinase
Solyc07g017230	652.40	9.74	-6.06	3.24E-237	Receptor like kinase
Solyc05g047570	120.41	1.63	-6.21	8.39E-43	Receptor like kinase
Solyc01g109530	821.61	12.50	-6.04	3.70E-82	Receptor like kinase
Solyc03g124050	105.00	1.83	-5.84	9.69E-40	Receptor like kinase
Solyc03g122230	10.07	0.53	-4.26	2.12E-05	Receptor like kinase
Solyc06g063360	570.29	45.87	-3.64	1.04E-42	Receptor like kinase
Solyc09g057680	30.89	10.64	-1.54	2.00E-19	Receptor like kinase
Solyc06g063150	194.30	32.99	-2.56	1.65E-11	Receptor like kinase
Solyc03g007050	4.94	49.07	3.31	2.56E-04	Receptor like kinase
Solyc03g093330	2.04	8.21	2.01	5.86E-04	Receptor like kinase
Solyc03g115610	2.84	8.86	1.64	1.79E-06	Receptor like kinase
Solyc01g067020	3.19	7.60	1.25	4.77E-03	Receptor like kinase
Solyc02g071820	30.31	150.54	2.31	2.82E-10	Receptor like kinase
Solyc02g081070	18.80	49.44	1.40	2.48E-03	Receptor like kinase
Solyc07g055810	37.52	109.49	1.55	6.60E-06	Receptor like kinase
Solyc07g056410	9.38	21.18	1.18	3.01E-05	Receptor like kinase
Solyc12g096710	15.33	38.69	1.34	1.66E-06	Receptor like kinase
Solyc02g068300	2.95	6.92	1.23	7.41E-04	Receptor like kinase
Solyc11g007400	15.08	35.40	1.23	3.08E-04	Receptor like kinase
Solyc12g049360	0.78	4.02	2.36	1.08E-05	Receptor like kinase
**CMF**					
Solyc12g096940	66.53402	4.152677	-4.00198	3.489E-09	CCT motif family protein

Supplemental table 7. The differentially expressed genes involved in calcium signaling in the pistil of WT and *SlNCED1*-OE-2 at stage 13-14.

Gene ID	RPKM WT	RPKM OE 2	Log2.Fold Change	p.value	Description
Solyc02g014560	97.76	24.96	-1.97	1.44E-03	Calcium binding atopy-related autoantigen
Solyc09g090590	219.68	20.83	-3.40	3.77E-16	Calcium binding protein Caleosin
Solyc10g076900	194.03	18.34	-3.40	1.01E-03	Calcium dependent protein kinase
Solyc10g076900	194.03	18.34	-3.40	8.64E-17	Calcium dependent protein kinase
Solyc10g081640	29.44	7.77	-1.92	3.71E-03	Calcium dependent protein kinase
Solyc01g006730	94.02	39.44	-1.25	8.60E-07	Calcium dependent protein kinase
Solyc12g011070	45.74	3.91	-3.55	7.75E-04	Calcium/proton exchanger
Solyc06g052040	61.63	1.32	-5.54	5.83E-08	Calcium-binding EF hand
Solyc07g065130	446.57	62.45	-2.84	3.86E-30	Calcium-binding protein
Solyc06g068700	531.61	64.13	-3.05	2.03E-72	Calcium-binding protein Calnexin
Solyc02g032820	184.33	8.57	-4.43	1.12E-22	Calcium-dependent protein kinase
Solyc11g064900	104.38	0.95	-6.78	2.59E-08	Calcium-dependent protein kinase
Solyc12g099790	455.40	12.17	-5.23	1.10E-38	Calcium-dependent protein kinase
Solyc06g073350	300.82	16.11	-4.22	8.38E-31	Calcium-dependent protein kinase
Solyc06g065380	201.22	26.04	-2.95	2.35E-21	Calcium-dependent protein kinase
Solyc01g008440	365.85	120.67	-1.60	1.48E-27	Calcium-dependent protein kinase
Solyc02g092450	241.99	60.63	-2.00	3.80E-44	Calcium-transporting ATPase
Solyc03g097100	73.93	18.88	-1.97	3.08E-14	Calmodulin
Solyc04g008000	61.11	11.22	-2.45	5.67E-10	Calmodulin
Solyc02g063340	97.99	1.33	-6.20	2.89E-11	Calmodulin 4
Solyc02g091480	155.23	7.21	-4.43	3.34E-19	Calmodulin 4
Solyc10g078650	116.75	0.89	-7.04	1.25E-11	Calmodulin-binding protein
Solyc04g050050	86.48	41.62	-1.06	8.08E-08	Calmodulin-binding protein
Solyc02g067220	103.79	4.00	-4.70	7.44E-12	Calmodulin-binding protein
Solyc05g050750	62.15	4.09	-3.92	7.88E-14	Calmodulin-binding protein
Solyc11g044920	48.58	3.59	-3.76	9.30E-09	Calmodulin-binding protein
Solyc01g005410	29.65	88.43	1.58	1.77E-05	Calcium binding protein Caleosin
Solyc06g006110	18.37	101.13	2.46	1.10E-07	Calcium/proton exchanger
Solyc08g007920	3.01	7.87	1.39	2.48E-19	Calmodulin binding protein
Solyc03g025510	30.68	75.70	1.30	6.04E-06	Calmodulin-binding heat shock protein
Solyc03g119250	31.47	97.45	1.63	4.76E-08	Calmodulin-binding protein
Solyc04g048900	172.32	447.69	1.38	1.49E-15	Calreticulin 2 calcium-binding protein
Solyc02g086990	141.83	29.34	-2.27	1.41E-16	Cyclic nucleotide gated channel (CNGC)
Solyc05g050360	3.73	19.22	2.37	1.53E-03	Cyclic nucleotide gated channel
Solyc03g007170	42.51	103.46	1.28	2.78E-05	FK506-binding protein

Supplemental table 8. The differentially expressed genes involved in small GTPase in the pistil of WT and *SlNCED1*-OE-2 at stage.

Gene ID	RPKM WT	RPKM OE 2	Log2.Fold Change	p.value	Description
Solyc02g083580	716.55	297.86	-1.27	3.09E-39	Ras GTPase
Solyc02g062020	742.35	353.01	-1.07	3.25E-31	Ras GTPase
Solyc03g006270	70.34	1.08	-6.03	3.70E-18	Rab GTPase
Solyc05g052900	31.80	1.31	-4.60	3.45E-06	Rab GTPase
Solyc06g076450	140.17	49.07	-1.51	7.80E-18	Rab GTPase
Solyc10g079030	18.98	3.06	-2.63	1.05E-03	Rab GTPase
Solyc12g005840	334.25	38.24	-3.13	1.14E-28	Rab GTPase
Solyc05g045660	265.01	1.74	-7.25	1.71E-17	Rab GTPase
Solyc05g051570	366.83	91.21	-2.01	2.23E-26	Rab GTPase
Solyc10g005340	61.03	15.16	-2.01	4.85E-05	Rop-interactive crib
Solyc09g072640	245.01	5.56	-5.46	1.26E-24	Rop-interactive crib
Solyc06g083800	213.81	51.19	-2.06	1.41E-14	Rop-interactive crib
Solyc06g008240	55.06	0.87	-5.99	2.25E-04	Rop-interactive crib
Solyc06g007780	29.67	66.87	1.17	4.14E-04	Ras GTPase
Solyc04g076140	558.30	138.67	-2.01	6.48E-24	Rho GTPase
Solyc01g005310	526.45	7.11	-6.21	1.86E-31	Dynamin like protein, GTPase region
Solyc12g098650	333.29	4.75	-6.13	3.18E-18	Rho GDP-dissociation inhibitor
Solyc04g076140	558.30	138.67	-2.01	6.48E-24	Rho GDP-dissociation inhibitor
Solyc11g012270	273.63	94.26	-1.54	2.50E-14	Rho GDP-dissociation inhibitor
Solyc05g054110	857.83	38.61	-4.47	1.14E-79	Rho GDP-dissociation inhibitor
Solyc04g014760	344.87	122.94	-1.49	8.40E-30	Rho GDP-dissociation inhibitor
Solyc08g067930	248.75	3.09	-6.33	1.42E-18	Arf GTPase activating protein
Solyc03g098500	500.84	15.99	-4.97	3.03E-34	Arf-GAP with GTPase

Supplemental table 9. The differentially expressed genes involved in lipid metabolism and transport in the pistil of WT and *SlNCED1*-OE-2 at stage 13-14.

Gene ID	RPKM WT	RPKM OE 2	Log2.Fold Change	p.value	Description
Solyc01g088430	27.73	330.74	3.58	1.47E-51	CER, Fatty acid hydroxylase
Solyc08g044260	48.68	142.39	1.55	3.34E-10	CER, Fatty acid hydroxylase
Solyc08g067410	5.12	13.73	1.42	1.11E-07	Fatty acid elongase 3-ketoacyl-CoA synthase
Solyc05g013220	20.04	46.63	1.22	3.67E-13	Fatty acid elongase 3-ketoacyl-CoA synthase
Solyc01g066620	2.60	155.12	5.90	1.85E-13	Fatty acid oxidation complex subunit alpha
Solyc03g051960	2.14	26.18	3.61	2.59E-04	Fatty acyl coA reductase
Solyc09g009580	3.30	56.77	4.10	4.21E-09	Fatty acyl coA reductase
Solyc11g067180	25.92	82.19	1.66	4.46E-05	Fatty acyl coA reductase
Solyc02g082910	3.60	9.39	1.38	3.34E-03	AMP-dependent synthetase and ligase
Solyc12g042460	1.73	20.10	3.54	2.32E-04	AMP-dependent synthetase and ligase
Solyc02g088710	1.35	17.13	3.67	3.89E-04	AMP-dependent synthetase and ligase
Solyc03g111170	45.25	116.85	1.37	3.77E-13	AMP-dependent synthetase and ligase
Solyc10g076200	1376.56	151.33	-3.19	3.50E-107	Non-specific lipid-transfer
Solyc06g059790	2496.16	429.20	-2.54	1.69E-151	Non-specific lipid-transfer
Solyc01g095780	2.35	60.59	4.69	8.40E-27	Non-specific lipid-transfer
Solyc12g006820	22.05	1.55	-3.83	6.97E-16	Fatty acid hydroxylase
Solyc02g022910	202.17	2.35	-6.43	1.50E-20	Fatty acid hydroxylase
Solyc04g010250	2260.80	731.13	-1.63	1.75E-169	Lipase-like protein
Solyc02g068070	14.12	43.03	1.61	9.86E-04	Lipase-like protein
Solyc08g078090	6.21	12.81	1.04	1.16E-05	Lipase-like protein
Solyc02g077100	6.03	15.94	1.40	1.70E-03	Lipase-like protein
Solyc03g118580	777.38	5.85	-7.05	8.14E-85	Lipid binding protein
Solyc06g009290	22.38	152.35	2.77	6.24E-28	Lipid binding protein
Solyc04g025400	247.29	2.87	-6.43	1.39E-17	Lipid phosphate phosphatase
Solyc05g043320	37.24	0.56	-6.05	4.07E-06	GDSL esterase/lipase
Solyc05g014210	202.55	5.88	-5.11	3.58E-39	GDSL esterase/lipase
Solyc05g043310	31.46	0.19	-7.39	2.81E-03	GDSL esterase/lipase
Solyc06g007490	31.79	2.19	-3.86	2.70E-03	GDSL esterase/lipase
Solyc04g081780	1.98	112.96	5.83	4.40E-20	GDSL esterase/lipase
Solyc05g013690	11.07	31.51	1.51	3.38E-03	GDSL esterase/lipase
Solyc03g097390	74.98	5.87	-3.68	6.91E-08	Acyl-ACP thioesterase
Solyc05g050090	597.10	29.73	-4.33	2.12E-129	Delta-6 fatty acid desaturase (ER6FAD)
Solyc07g040740	181.52	85.34	-1.09	3.41E-06	Diacylglycerol acyltransferase (DGAT)
Solyc01g009870	87.04	32.28	-1.43	2.02E-07	Glycerophosphodiester phosphodiesterase
Solyc02g014490	218.51	3.19	-6.10	2.40E-22	Glycerophosphoryl diester
Solyc02g014530	253.60	9.94	-4.67	7.82E-16	Glycerophosphoryl diester
Solyc06g007140	124.15	5.72	-4.44	6.89E-08	Omega-3 fatty acid desaturase 3 (LeFAD3)
Solyc06g007130	2804.96	533.29	-2.40	5.44E-182	Omega-3 fatty acid desaturase 3

Solyc03g058430	153.65	36.20	-2.09	1.44E-78	Omega-3 fatty acid desaturase 6 (LeFAD6)
Solyc07g006100	66.97	26.87	-1.32	8.67E-05	Oxysterol-binding protein (OSBP)
Solyc10g081800	63.87	13.28	-2.27	8.36E-04	Phosphatidylglycerol (PITP)
Solyc09g090350	24.90	65.04	1.39	4.32E-16	Long-chain fatty alcohol dehydrogenase
Solyc06g062870	77.37	1.14	-6.08	1.35E-13	Phospholipid diacylglycerol acyltransferase
Solyc07g052480	425.59	1348.47	1.66	0.00E+00	Isocitrate lyase (ICL)
Solyc01g099190	26.51	0.73	-5.18	1.80E-05	Lipoxygenase (LOX)
Solyc12g011040	10.96	38.37	1.81	4.57E-05	Lipoxygenase

Supplemental Table 10. The differentially expressed genes involved in sugar metabolism and transport in the pistil of WT and *SlNCED1*-OE-2 at stage 13-14.

Gene ID	RPKM WT	RPKM OE 2	Log2.Fold Change	p.value	Description
Solyc09g092130	1194.21	242.47	-2.30	1.97E-129	Sucrose phosphate synthase
Solyc08g042000	352.10	62.30	-2.50	1.50E-37	Sucrose phosphate synthase
Solyc06g071400	3364.09	85.56	-5.30	0.00E+00	SWEET sucrose transporter
Solyc04g064620	59.15	11.23	-2.40	1.30E-04	SWEET sucrose transporter
Solyc11g017010	150.20	370.61	1.30	1.59E-46	Sucrose transporter
Solyc02g062860	41.46	1.10	-5.24	2.94E-15	Sugar transporter
Solyc02g062870	34.23	0.97	-5.15	6.23E-15	Sugar transporter
Solyc01g008240	2116.88	22.35	-6.57	0.00E+00	Sugar transporter
Solyc06g054270	3109.48	249.71	-3.64	0.00E+00	Sugar transporter
Solyc12g010990	83.79	3.47	-4.60	5.80E-07	Sugar transporter
Solyc10g079910	1210.59	91.72	-3.72	1.00E-132	Sugar transporter
Solyc11g012880	69.82	12.79	-2.45	2.09E-06	Sugar transporter
Solyc01g010530	0.46	7.39	3.99	4.45E-08	Sugar transporter
Solyc03g006650	10.41	42.46	2.03	1.15E-08	Sugar transporter
Solyc08g079080	596.59	50.92	-3.55	3.60E-84	Acid beta-fructofuranosidase
Solyc03g070390	228.79	8.65	-4.72	1.86E-36	Alpha-1 4-glucan-protein synthase
Solyc02g065740	646.61	5.49	-6.88	3.47E-90	Alpha-1 4-glucan-protein synthase
Solyc09g010090	5310.54	637.10	-3.06	0.00E+00	Beta-fructofuranosidase insoluble
Solyc10g085650	15.15	64.97	2.10	1.04E-10	Beta-fructofuranosidase insoluble isoenzyme
Solyc10g083290	23.48	65.53	1.48	7.82E-07	Beta-fructofuranosidase insoluble isoenzyme
Solyc05g005820	4835.67	1102.60	-2.13	3.03E-172	Glyceraldehyde-3-phosphate dehydrogenase
Solyc02g091830	196.93	71.81	-1.46	1.71E-11	Hexokinase (HXK)
Solyc03g122130	7.62	2.77	-1.46	9.32E-09	L-lactate dehydrogenase (LDH)
Solyc10g007600	89.44	245.76	1.46	1.03E-09	L-lactate dehydrogenase (LDH)
Solyc08g066100	199.23	20.69	-3.27	4.29E-12	Phosphofructokinase family protein (PFK)
Solyc01g008790	48.40	123.30	1.35	1.56E-11	Phosphofructokinase family protein (PFK)
Solyc12g098930	157.35	37.29	-2.08	1.48E-10	Pyruvate dehydrogenase kinase (PDK)
Solyc09g00884	596.87	223.74	-1.42	1.50E-20	Pyruvate kinase (PK)
Solyc08g077180	435.72	158.46	-1.46	9.08E-24	Pyruvate kinase (PK)
Solyc10g049890	325.05	130.21	-1.32	1.25E-43	Phosphoglycerate dehydrogenase (PHGDH)
Solyc07g065980	35.47	125.21	1.82	1.99E-11	Alkaline alpha galactosidase I
Solyc03g007950	35.48	6.12	-2.54	5.77E-05	Glycoside hydrolase family 28 protein

Supplemental table 11. The differentially expressed genes involved in cytoskeleton in the pistil of WT and *SlNCED1*-OE-2 at stage 13-14.

Gene ID	RPKM WT	RPKM OE 2	Log2.Fold Change	p.value	Description
Solyc06g076090	1617.48	43.07	-5.23	1.20E-279	Actin
Solyc05g054480	1109.61	100.16	-3.47	3.38E-145	Actin
Solyc11g065990	569.38	131.28	-2.12	7.92E-79	Actin
Solyc10g086460	1549.02	211.58	-2.87	1.52E-183	Actin
Solyc03g025750	236.26	3.73	-5.98	2.00E-21	Actin depolymerizing factor
Solyc01g094400	334.09	10.52	-4.99	2.73E-39	Actin depolymerizing factor
Solyc10g017550	217.87	2.19	-6.64	2.14E-22	Actin depolymerizing factor
Solyc06g069670	49.49	22.39	-1.14	4.87E-03	Formin
Solyc05g046120	107.03	36.49	-1.55	3.76E-17	Formin
Solyc07g064010	67.62	32.38	-1.06	1.65E-08	Formin
Solyc07g049600	283.68	92.93	-1.61	3.08E-14	Formin
Solyc11g044740	926.71	13.40	-6.11	1.68E-48	LIM
Solyc08g007940	752.93	7.38	-6.67	8.88E-66	LIM
Solyc10g079400	330.43	27.68	-3.58	4.36E-20	Gelsolin
Solyc01g100120	786.80	25.93	-4.92	2.14E-72	Kinesin
Solyc04g081060	507.26	17.13	-4.89	4.59E-59	Kinesin
Solyc02g062330	232.97	48.45	-2.27	3.65E-19	Kinesin
Solyc12g099540	218.46	91.14	-1.26	2.31E-12	Kinesin
Solyc12g036810	77.85	11.03	-2.82	4.42E-06	Kinesin
Solyc06g069130	191.71	56.26	-1.77	1.00E-08	Kinesin
Solyc12g088830	283.29	14.62	-4.28	3.16E-114	Myosin heavy chain-like
Solyc04g015110	44.99	1.97	-4.52	1.39E-21	Myosin heavy chain-like protein
Solyc06g083960	254.23	31.01	-3.04	8.90E-15	Myosin XI
Solyc06g008530	720.74	95.75	-2.91	6.94E-37	Myosin XI
Solyc11g006220	594.68	120.82	-2.30	7.07E-27	Myosin XI
Solyc09g091080	173.89	61.99	-1.49	1.44E-06	Myosin XI-F

Supplemental Table 12. The differentially expressed genes involved in cell wall metabolism in the pistil of WT and *SlNCED1*-OE-2 at stage 13-14.

Gene ID	RPKM WT	RPKM OE 2	Log2.Fold Change	p.value	Description
Solyc01g111540	3460.18	22.64	-7.26	2.67E-215	Beta-galactosidase
Solyc10g055470	26853.71	4207.01	-2.67	0.00E+00	Beta-galactosidase
Solyc12g008840	169.68	446.16	1.39	2.24E-17	Beta-galactosidase
Solyc02g078950	45.27	110.59	1.29	1.59E-05	Beta-galactosidase
Solyc07g042220	24.45	157.48	2.69	1.14E-14	Beta-galactosidase
Solyc06g062660	130.63	349.10	1.42	7.24E-15	Beta-galactosidase
Solyc10g074620	384.39	5.49	-6.13	3.66E-98	Cellulose synthase-like
Solyc01g067520	46.38	1.31	-5.15	3.79E-13	Cellulose synthase-like
Solyc12g088240	48.34	1.04	-5.54	8.11E-10	Cellulose synthase-like
Solyc05g053560	127.06	0.96	-7.04	1.77E-28	Cellulose synthase-like
Solyc08g006300	153.89	2.57	-5.90	2.12E-42	Fasciclin-like arabinogalactan protein
Solyc08g006810	316.81	116.50	-1.44	4.28E-94	Fasciclin-like arabinogalactan protein
Solyc05g054440	1803.57	13.33	-7.08	0.00E+00	Glucan endo-1 3-beta-glucosidase
Solyc03g058450	335.08	3.01	-6.80	4.65E-03	Glucan endo-1 3-beta-glucosidase
Solyc05g015170	59.95	1.62	-5.21	1.14E-12	Glucan endo-1 3-beta-glucosidase
Solyc05g015160	143.20	1.71	-6.39	1.87E-35	Glucan endo-1 3-beta-glucosidase
Solyc04g011730	48.20	0.62	-6.27	6.57E-14	Glucan endo-1 3-beta-glucosidase
Solyc04g051590	166.01	2.31	-6.17	1.18E-41	Glucan endo-1 3-beta-glucosidase
Solyc04g011720	25.16	0.45	-5.80	1.90E-08	Glucan endo-1 3-beta-glucosidase
Solyc11g005980	8062.31	126.22	-6.00	0.00E+00	Glucan endo-1 3-beta-glucosidase
Solyc01g010310	25.66	66.58	1.38	1.36E-04	Glucan endo-1 3-beta-glucosidase
Solyc07g056310	5.74	21.75	1.92	1.63E-03	Glucan endo-1 3-beta-glucosidase
Solyc03g058910	2987.99	47.85	-5.96	0.00E+00	Pectate lyase
Solyc06g071020	34.42	2.07	-4.05	4.35E-07	Pectate lyase
Solyc03g058890	677.14	8.81	-6.26	1.40E-256	Pectate lyase
Solyc03g113150	30.70	7.60	-2.01	4.00E-05	Pectate lyase
Solyc05g007080	1251.01	9.46	-7.05	2.05E-243	Pectate lyase
Solyc02g087670	2302.96	20.92	-6.78	1.30E-09	Pectate lyase
Solyc02g067450	4900.49	53.33	-6.52	0.00E+00	Pectate lyase
Solyc01g010740	65.42	2.15	-4.93	4.74E-14	Pectate lyase
Solyc03g078260	9590.63	145.98	-6.04	0.00E+00	Pectinacetylesterase
Solyc12g005320	22376.02	3279.63	-2.77	0.00E+00	Pectinacetylesterase
Solyc08g075030	186.56	6.00	-4.96	4.61E-12	Pectinacetylesterase
Solyc06g084620	1178.28	21.41	-5.78	0.00E+00	Pectinesterase
Solyc01g099940	283.89	4.84	-5.88	6.29E-82	Pectinesterase
Solyc07g017560	509.41	5.61	-6.51	1.08E-180	Pectinesterase
Solyc01g057220	20.33	0.31	-6.02	6.36E-09	Pectinesterase
Solyc01g068120	790.96	7.64	-6.69	5.95E-284	Pectinesterase

Solyc01g066360	675.43	6.74	-6.65	0.00E+00	Pectinesterase
Solyc01g066420	477.95	7.51	-5.99	0.00E+00	Pectinesterase
Solyc05g054360	299.75	5.33	-5.81	1.95E-67	Pectinesterase
Solyc12g099410	914.96	16.34	-5.81	7.22E-178	Pectinesterase
Solyc12g099230	62.56	0.42	-7.23	7.72E-06	Pectinesterase
Solyc05g052110	549.66	5.54	-6.63	1.09E-68	Pectinesterase
Solyc05g052120	591.11	5.97	-6.63	7.27E-53	Pectinesterase
Solyc03g078090	45.80	22.83	-1.00	3.74E-05	Pectinesterase
Solyc01g059940	38.20	1.25	-4.93	1.02E-19	Pectinesterase
Solyc01g091050	175.69	471.53	1.42	1.60E-146	Pectinesterase
Solyc08g076080	55.82	19.17	-1.54	8.64E-07	Xyloglucan endotransglucosylase/hydrolase
Solyc07g009380	1.84	14.10	2.94	3.09E-04	Xyloglucan endotransglucosylase/hydrolase
Solyc08g060970	24.38	63.17	1.37	2.53E-03	Polygalacturonase
Solyc03g113230	4.15	45.38	3.45	1.29E-04	Polygalacturonase
Solyc06g009200	207.88	3.27	-5.99	4.14E-27	Polygalacturonase
Solyc01g066070	149.28	2.30	-6.02	1.29E-22	Polygalacturonase
Solyc07g044870	2110.54	11.45	-7.53	2.57E-296	Polygalacturonase
Solyc07g015870	139.35	1.08	-7.02	2.88E-13	Polygalacturonase
Solyc06g005560	27.84	116.81	2.07	5.68E-21	Expansin
Solyc08g077330	15.49	51.38	1.73	1.68E-04	Expansin-like protein
Solyc06g066120	43.59	0.14	-8.25	2.38E-05	Endoglucanase (EG)
Solyc09g075360	119.24	333.10	1.48	1.29E-28	Endoglucanase
Solyc10g081670	655.23	7.60	-6.43	5.18E-48	Pectinesterase inhibitor
Solyc10g074920	147.39	656.34	2.15	3.13E-69	Mannan endo-1 4-beta-mannosidase
Solyc04g080620	21.78	12.15	-0.84	4.03E-03	Mannan endo-1 4-beta-mannosidase
Solyc03g046200	1.69	12.41	2.88	8.08E-18	Endo-1 3-beta-glucanase
Solyc08g081970	88.17	1.77	-5.63	2.42E-14	Arabinogalactan peptide（AGP）
Solyc02g032910	2.11	17.47	3.05	8.90E-06	Glycine rich protein（GRP)
Solyc09g092710	12.42	57.45	2.21	9.65E-12	Glycine rich protein
Solyc08g078650	10.62	67.17	2.66	9.05E-14	Glycosyl transferase family 8 glycogenin
Solyc12g056200	3.79	16.89	2.16	1.18E-04	Glycosyltransferase-like protein
Solyc04g014430	0.53	50.40	6.58	4.86E-05	Rhamnogalacturonate lyase（RGL)
Solyc04g076650	28.16	174.83	2.63	1.43E-12	Rhamnogalacturonate lyase
Solyc04g076640	19.51	76.98	1.98	6.48E-05	Rhamnogalacturonate lyase

Supplemental Table 13. The differentially expressed genes involved in stress responses in the pistil of WT and *SlNCED1*-OE-2 at stage 13-14.

Gene ID	RPKM WT	RPKM OE 2	Log2.Fold Change	p.value	Description
**Dehydration**					
Solyc06g051000	130.45	0.03	-12.32	2.95E-16	Dehydration-responsive family protein
Solyc02g084670	1715.58	107.58	-4.00	8.51E-276	Dehydration-responsive family protein
Solyc01g009600	136.66	48.31	-1.50	4.56E-14	Dehydration-responsive family protein
Solyc08g013740	319.36	21.23	-3.91	1.81E-55	Dehydration-responsive family protein
Solyc06g069870	185.15	15.40	-3.59	1.79E-28	Dehydration-responsive family protein
Solyc04g077400	87.58	34.35	-1.35	1.90E-05	Dehydration-responsive family protein
Solyc02g084840	7.32	29.11	1.99	2.31E-04	Dehydrin DHN1
**Heat stress**					
Solyc03g113930	13.44	4.64	-1.53	4.34E-03	class IV heat shock protein
Solyc11g044450	72.50	31.05	-1.22	1.52E-10	Heat shock protein DnaJ
Solyc10g007240	136.83	22.84	-2.58	3.46E-14	Heat shock protein DnaJ
Solyc04g055000	22.82	4.45	-2.36	5.24E-04	Heat shock protein DnaJ
Solyc03g025510	30.68	75.70	1.30	6.04E-06	Calmodulin-binding heat shock protein
**Cold stress**					
Solyc01g109170	7.38	22.79	1.63	2.70E-04	Cold acclimation protein
**Salt stress**					
Solyc03g006110	24.03	1.17	-4.36	3.20E-10	CBL-interacting protein kinase
**Heavy metal stress**					
Solyc10g086280	172.19	487.99	1.50	2.60E-32	Heavy metal-associated domain
**Disease stress**					
Solyc10g085960	23.14	6.66	-1.80	7.23E-08	Pathogenesis-related protein
Solyc01g106600	81.94	1.51	-5.76	1.08E-230	Pathogenesis-related protein
Solyc04g064880	29.85	133.52	2.16	5.53E-16	Pathogenesis-related protein-like protein
**Osmotic stress**					
Solyc08g080670	8.82	39.89	2.18	1.90E-14	Osmotin-like protein
Solyc08g080650	13.81	63.73	2.21	3.54E-18	Osmotin-like protein
Solyc08g080660	4.27	30.17	2.82	6.93E-12	Osmotin-like protein
Solyc08g080640	14.85	116.31	2.97	1.53E-47	Osmotin-like protein
**ROS scavenging**					
Solyc01g081250	139.59	63.01	-1.15	8.06E-12	Glutathione-S-transferase
Solyc05g006230	20.82	0.58	-5.16	2.91E-10	Peroxidase
Solyc01g007950	835.00	127.00	-2.72	3.99E-109	Peroxidase
Solyc05g013350	37.64	0.54	-6.12	2.48E-14	Peroxidase
Solyc08g007150	154.39	4.46	-5.11	1.23E-67	Peroxidase
Solyc04g080760	11.29	97.81	3.12	4.88E-19	Peroxidase
Solyc07g017880	12.62	66.11	2.39	3.07E-10	Peroxidase
Solyc04g071900	88.01	285.53	1.70	9.25E-41	Peroxidase

Solyc10g076240	1.05	35.21	5.06	1.59E-08	Peroxidase
Solyc11g018800	11.26	38.33	1.77	8.05E-04	Peroxidase
Solyc04g071890	135.16	387.26	1.52	3.43E-51	Peroxidase
Solyc01g058520	4.96	58.20	3.55	1.45E-15	Peroxidase
**Others**					
Solyc10g011900	31.38	0.51	-5.95	4.96E-03	Late embryogenesis abundant protein
Solyc09g061960	620.20	4.41	-7.13	1.93E-68	Late embryogenesis abundant protein
Solyc05g053450	526.08	7.52	-6.13	1.15E-41	Late embryogenesis abundant protein
Solyc01g097960	192.49	1.71	-6.81	4.07E-27	Late embryogenesis abundant protein
Solyc09g091040	32.68	1.19	-4.78	7.24E-04	Late embryogenesis abundant protein
Solyc02g085150	1206.87	258.65	-2.22	3.68E-202	LEA-like protein
Solyc10g078760	382.44	1.10	-8.44	4.85E-17	Seed maturation protein LEA 4
Solyc10g078770	14.11	77.82	2.46	9.92E-06	Seed maturation protein LEA 4
Solyc05g054270	174.51	1.29	-7.09	1.40E-14	Universal stress protein
Solyc01g066810	3356.44	27.44	-6.93	0.00E+00	Universal stress protein
Solyc12g099520	58.54	0.58	-6.66	1.17E-04	Universal stress protein
Solyc10g086670	222.38	1.12	-7.63	3.95E-11	Universal stress protein
Solyc09g090850	21.73	0.68	-5.00	2.11E-06	Universal stress protein
Solyc06g053380	110.37	24.93	-2.15	8.90E-28	Chitinase (Chi)
Solyc10g055800	89.20	954.53	3.42	5.90E-197	Chitinase
Solyc01g097270	29.13	125.98	2.11	5.91E-15	Chitinase
Solyc10g055810	13.81	53.55	1.95	1.80E-05	Endochitinase
Solyc11g011340	209.38	68.85	-1.60	9.22E-16	Alcohol dehydrogenase (ADH)
Solyc04g082170	192.51	6.68	-4.85	5.77E-11	Alcohol dehydrogenase
Solyc04g082180	291.52	13.96	-4.38	4.45E-21	Alcohol dehydrogenase
Solyc04g064710	131.17	38.51	-1.77	3.52E-06	Alcohol dehydrogenase
Solyc03g096550	8.84	27.59	1.64	1.09E-04	Wound/stress protein
Solyc10g085920	267.58	40.02	-2.74	2.36E-20	Bifunctional polymyxin resistance protein ArnA
Solyc12g099480	1790.11	12.13	-7.21	3.30E-109	Bifunctional polymyxin resistance protein ArnA
Solyc01g066700	696.80	69.13	-3.33	1.48E-39	Bifunctional polymyxin resistance protein ArnA
Solyc12g032930	52.52	4.11	-3.67	1.48E-03	CBS domain containing protein
Solyc03g019680	1034.13	209.58	-2.30	8.38E-66	CBS domain containing protein
Solyc12g035710	326.85	68.76	-2.25	1.02E-14	CBS domain containing protein
Solyc10g009010	47.40	1.02	-5.54	5.89E-16	CBS domain containing protein
Solyc03g006470	204.09	1.90	-6.74	6.95E-64	CBS domain containing protein
Solyc03g025250	65.43	8.10	-3.01	8.63E-08	Multidrug resistance protein mdtK
Solyc08g080310	16.68	0.38	-5.44	2.92E-08	Multidrug resistance protein mdtK
Solyc10g007380	71.39	15.83	-2.17	3.51E-06	Multidrug resistance protein mdtK
Solyc10g007370	207.64	91.14	-1.19	4.27E-12	Multidrug resistance protein mdtK
Solyc07g006740	23.58	1.12	-4.39	1.44E-03	Multidrug resistance protein mdtK
Solyc06g075470	66.63	30.51	-1.13	2.26E-04	Nucleoside diphosphate kinase (NDPK)
Solyc08g008340	573.43	256.17	-1.16	9.24E-19	Nucleoside diphosphate kinase
Solyc06g009540	20.85	0.26	-6.34	1.75E-24	Pto-like, resistance protein
Solyc01g109950	49.65	1.82	-4.77	3.50E-70	Pto-like, resistance protein

Solyc07g008400	7.19	2.37	-1.60	2.19E-08	Pto-like, resistance protein
Solyc11g020230	3.08	8.65	1.49	3.53E-03	Pto-like, resistance protein
Solyc02g089090	25.16	61.56	1.29	4.73E-17	Pto-like, resistance protein
Solyc04g007070	24.50	51.96	1.08	2.91E-15	Cc-nbs-lrr, resistance protein
Solyc04g007060	13.78	27.91	1.02	3.45E-10	Cc-nbs-lrr, resistance protein
Solyc04g008200	5.47	12.83	1.23	1.51E-04	Cc-nbs-lrr, resistance protein
Solyc12g094660	12.74	26.09	1.03	7.24E-11	Cc-nbs-lrr, resistance protein
Solyc01g087200	5.76	12.82	1.15	1.39E-03	Cc-nbs-lrr, resistance protein
Solyc05g012890	2.21	6.94	1.65	1.34E-03	Cc-nbs-lrr, resistance protein
Solyc04g007320	2.66	9.60	1.85	1.83E-03	Tir-nbs-lrr, resistance protein
Solyc01g008800	8.74	25.36	1.54	9.96E-07	Tir-nbs-lrr, resistance protein
Solyc12g017480	21.50	6.29	-1.77	2.34E-03	Lrr, resistance protein
Solyc05g012420	789.58	122.62	-2.69	7.97E-43	14-3-3 protein
Solyc01g010360	892.21	8.03	-6.80	4.98E-53	14-3-3-like protein

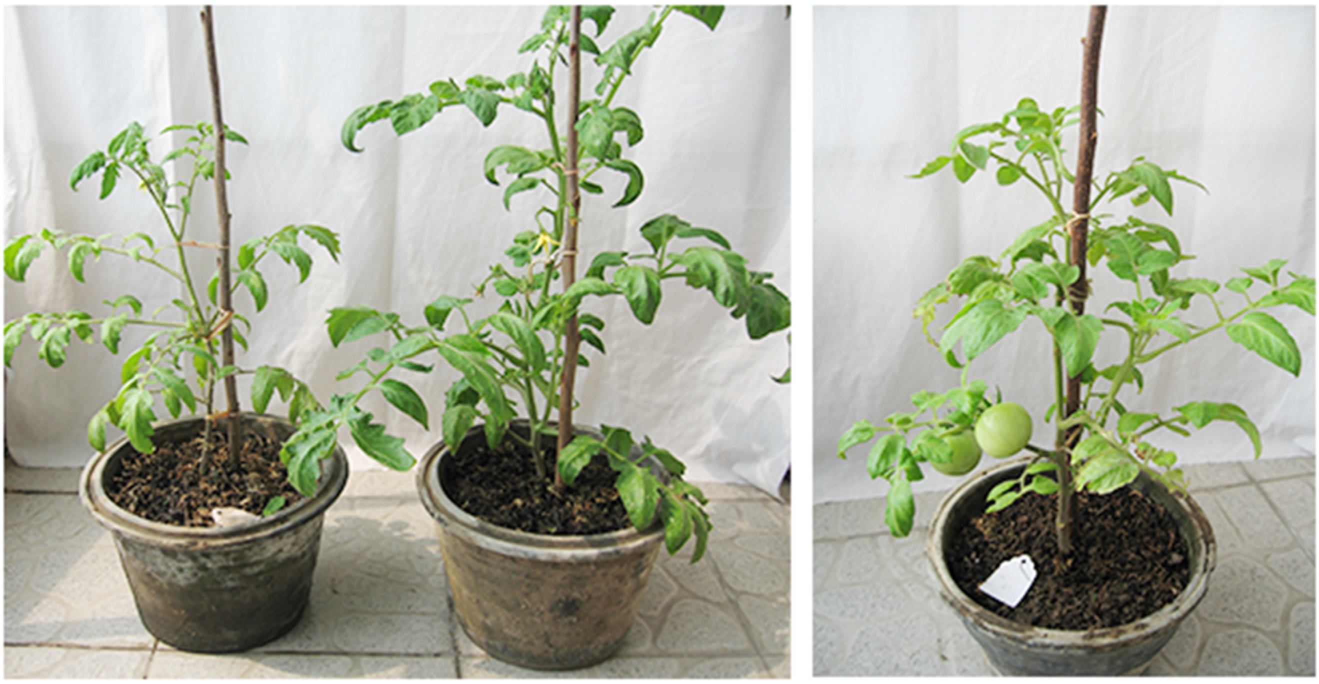

WT RNAi OE

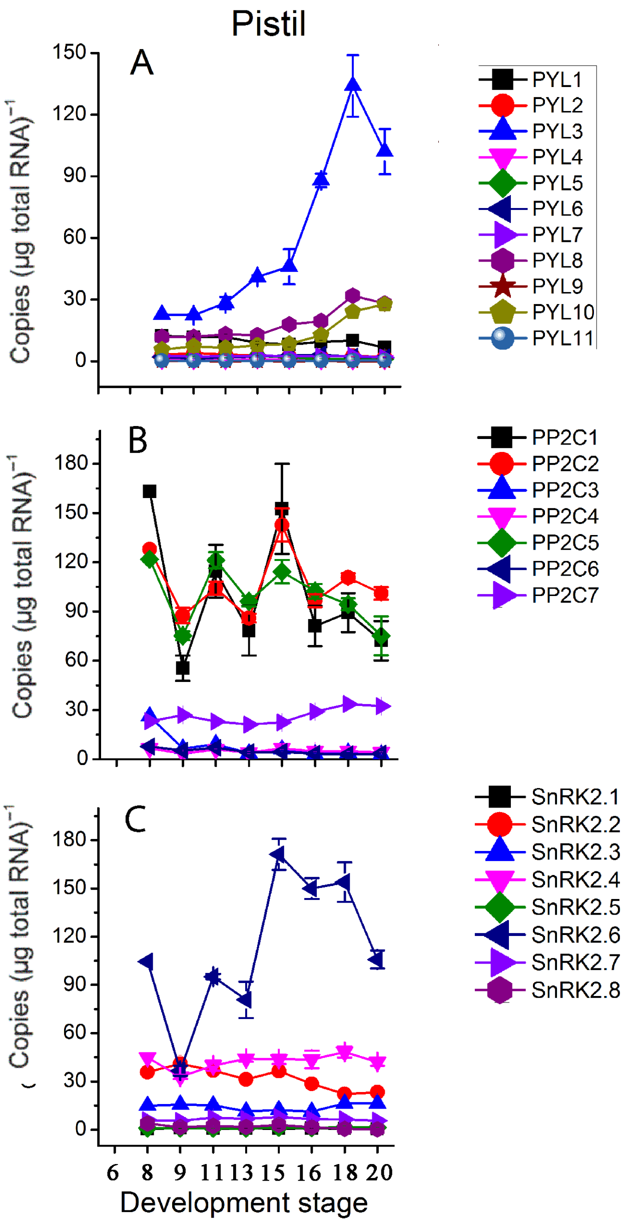
Pistil
A
B
C
Copies (μg total RNA)⁻¹
150
120
90
60
30
0
180
Development stage
6
8
9
11
13
15
16
18
20
PYL1
PYL2
PYL3
PYL4
PYL5
PYL6
PYL7
PYL8
PYL9
PYL10
PYL11
PP2C1
PP2C2
PP2C3
PP2C4
PP2C5
PP2C6
PP2C7
SnRK2.1
SnRK2.2
SnRK2.3
SnRK2.4
SnRK2.5
SnRK2.6
SnRK2.7
SnRK2.8

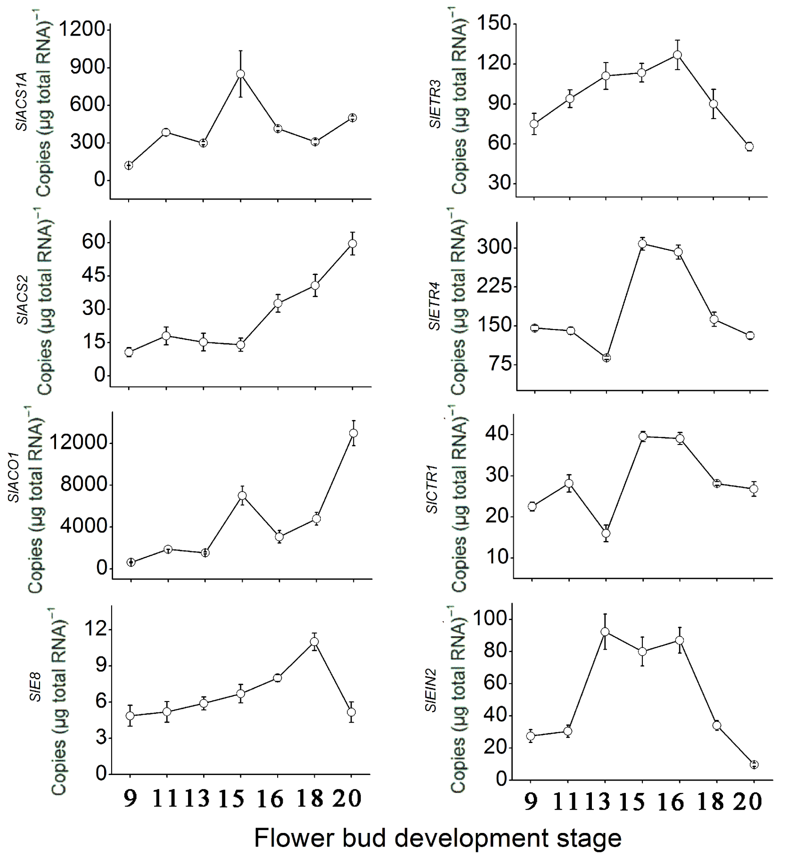

SlACS1A
SlETR3
SlACS2
SlETR4
SlACO1
SlCTR1
SlE8
SlEIN2
Copies (μg total RNA)⁻¹
9 11 13 15 16 18 20
Flower bud development stage

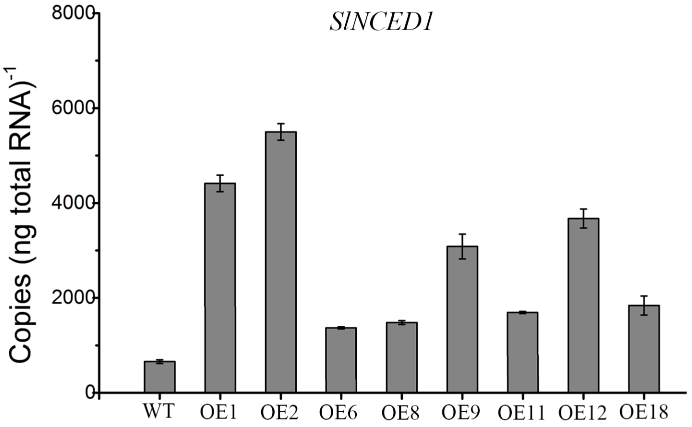
SlNCED1
Copies (ng total RNA)-1
8000
6000
4000
2000
0
WT
OE1
OE2
OE6
OE8
OE9
OE11
OE12
OE18

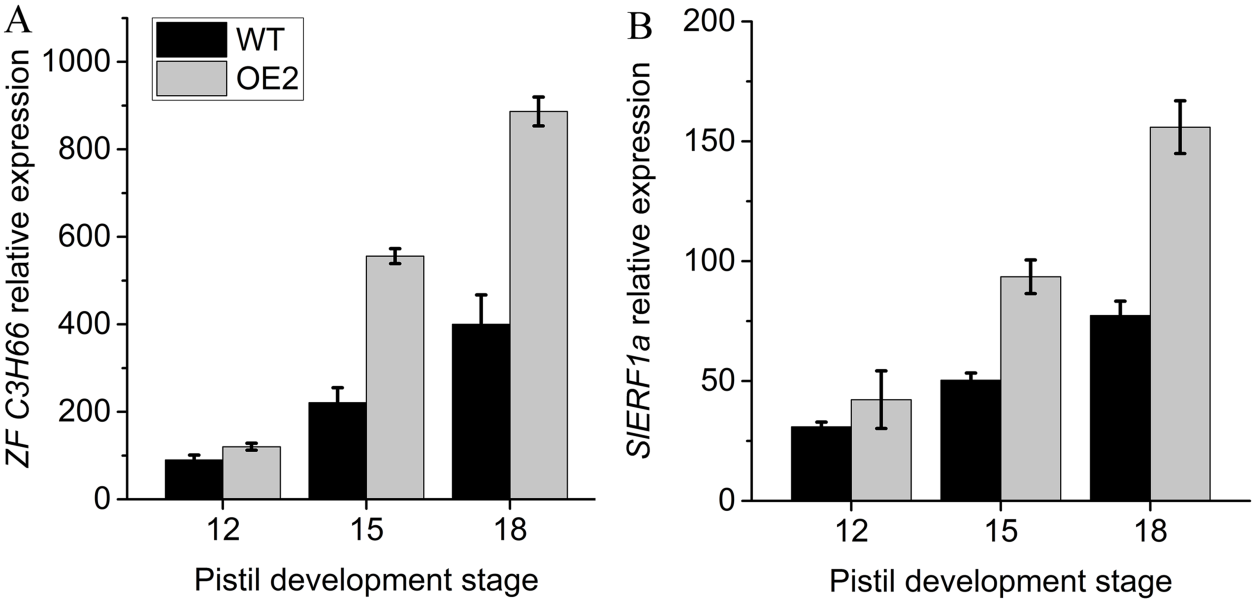
A
B
WT
OE2
ZF C3H66 relative expression
SlERF1a relative expression
0
200
400
600
800
1000
0
50
100
150
200
12
15
18
Pistil development stage
Pistil development stage

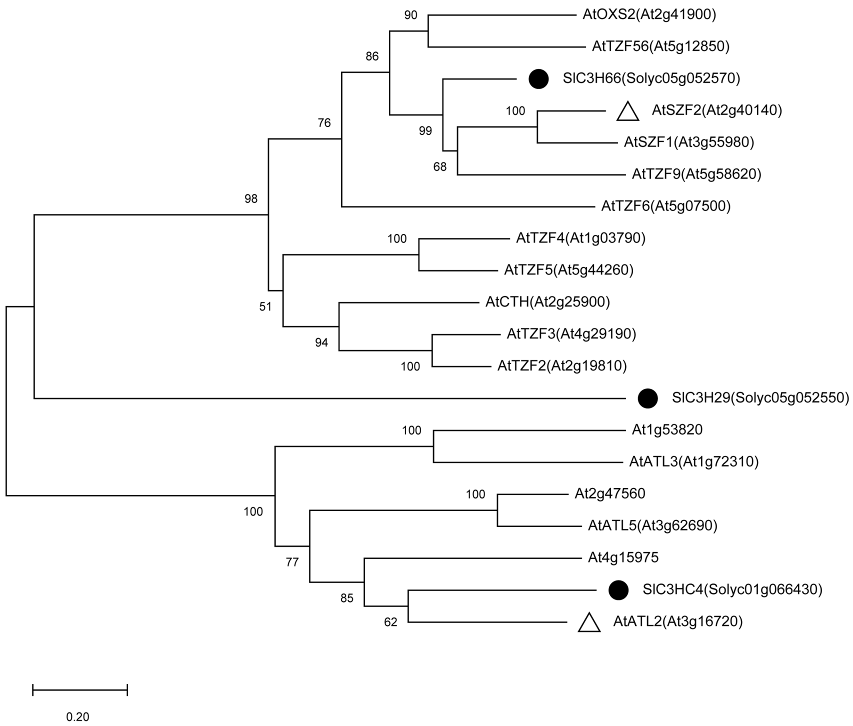
AtOXS2(At2g41900)
AtTZF56(At5g12850)
SlC3H66(Solyc05g052570)
AtSZF2(At2g40140)
AtSZF1(At3g55980)
AtTZF9(At5g58620)
AtTZF6(At5g07500)
AtTZF4(At1g03790)
AtTZF5(At5g44260)
AtCTH(At2g25900)
AtTZF3(At4g29190)
AtTZF2(At2g19810)
SlC3H29(Solyc05g052550)
At1g53820
AtATL3(At1g72310)
At2g47560
AtATL5(At3g62690)
At4g15975
SlC3HC4(Solyc01g066430)
AtATL2(At3g16720)
90
86
76
98
100
99
68
100
51
94
100
100
100
100
77
85
62
0.20

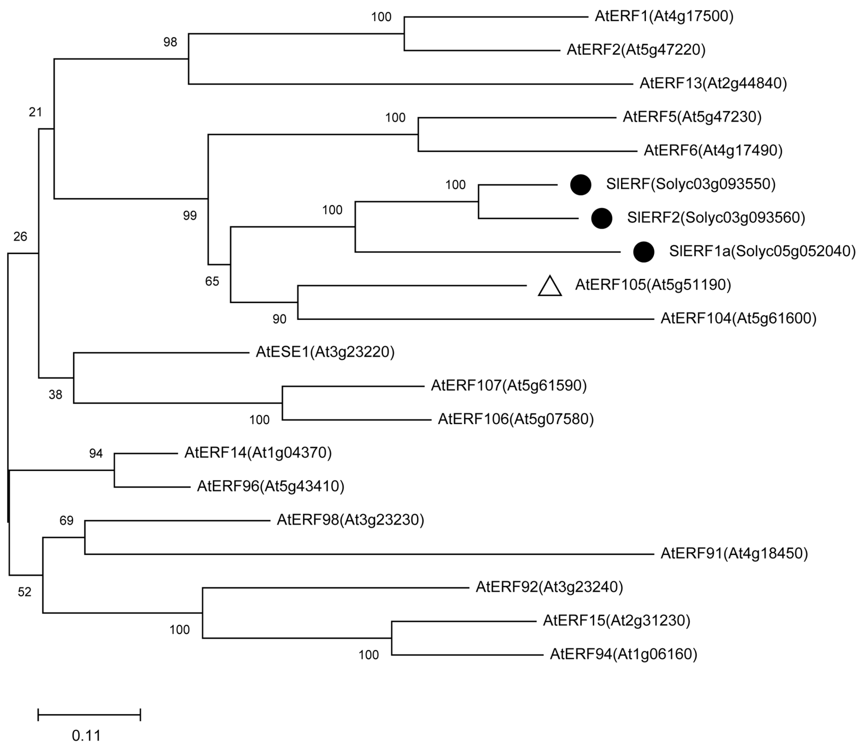

AtERF1(At4g17500)
AtERF2(At5g47220)
AtERF13(At2g44840)
AtERF5(At5g47230)
AtERF6(At4g17490)
SlERF(Solyc03g093550)
SlERF2(Solyc03g093560)
SlERF1a(Solyc05g052040)
AtERF105(At5g51190)
AtERF104(At5g61600)
AtESE1(At3g23220)
AtERF107(At5g61590)
AtERF106(At5g07580)
AtERF14(At1g04370)
AtERF96(At5g43410)
AtERF98(At3g23230)
AtERF91(At4g18450)
AtERF92(At3g23240)
AtERF15(At2g31230)
AtERF94(At1g06160)
100
98
21
100
100
99
100
65
90
26
38
100
94
69
52
100
100
0.11

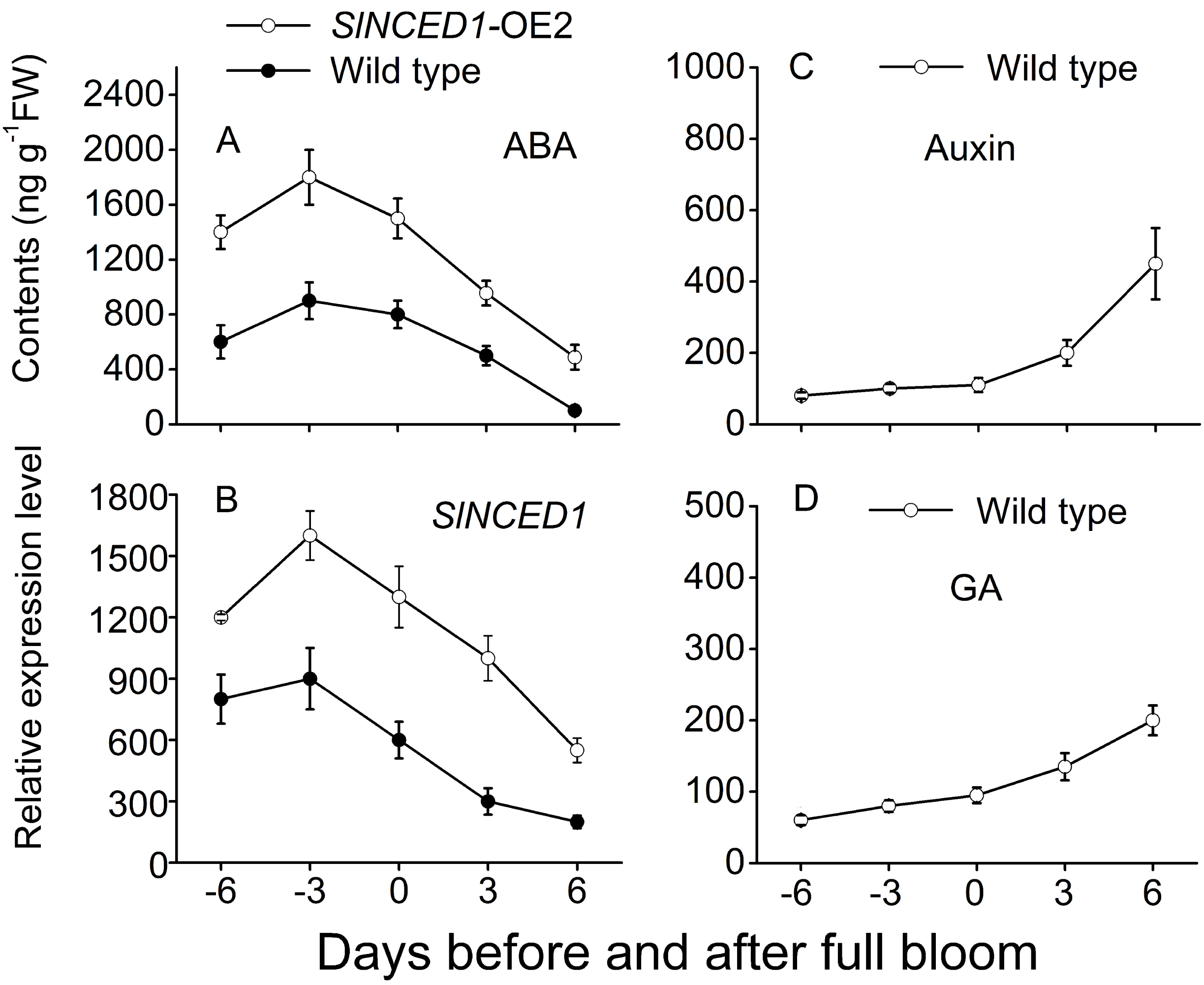
SlNCED1-OE2
Wild type
Contents (ng g-1 FW)
A
ABA
2400
2000
1600
1200
800
400
0
C
Wild type
Auxin
1000
800
600
400
200
0
Relative expression level
B
SlNCED1
1800
1500
1200
900
600
300
0
D
Wild type
GA
500
400
300
200
100
0
-6
-3
0
3
6
Days before and after full bloom
